# Identifying underweight in infants and children using growth charts, lookup tables and a novel “MAMI” slide chart: A cross-over diagnostic and acceptability study

**DOI:** 10.1371/journal.pgph.0002303

**Published:** 2023-08-30

**Authors:** Meenakshi Monga, Catherine Sikorski, Himali de silva, Marie McGrath, Marko Kerac

**Affiliations:** 1 Department of Population Health, London School of Hygiene and Tropical Medicine, London, United Kingdom; 2 Guy’s and St Thomas’ NHS Foundation Trust, London, United Kingdom; 3 Emergency Nutrition Network, Oxford, United Kingdom; McGill University, CANADA

## Abstract

Malnutrition is a leading cause of preventable deaths in infants and children. To benefit from treatment and prevention programmes, malnourished children must first be identified. Low weight-for-age is an anthropometric indicator of malnutrition which is gaining much recent attention because it is particularly effective at identifying children at highest risk of death. However, assessing weight-for-age can be challenging. We aimed to evaluate a novel, low-cost weight-for-age slide chart and compare its performance against two traditional methods. We conducted a cross-over diagnostic study comparing a new “MAMI” slide-chart against traditional growth charts and look-up tables. Participants were health and public health professionals working or studying in the UK. Each acted as their own control, using all three methods but in random order. Under timed conditions, they evaluated hypothetical scenarios, arranged in a random sequence. Each tool’s diagnostic accuracy and response rate were compared. User preferences were also recorded. Sixty-two participants took part. Diagnostic accuracy was highest for the MAMI chart: 79%(351/445) correct assessments. Accuracy using look-up tables was 70%(308/438). Growth charts performed worst: 61%(217/353) correct (p-value<0.01). The mean number of scenarios (±SD) correctly identified by each participant in 4-minutes was 3.5(±2.19) using growth charts; 4.97(±2.50) using look-up tables; 5.66(±2.69) using MAMI charts (ANOVA, p-value<0.01). This translates to approximately 53, 75 and 85 correct assessments per participant in an hour for the respective tools. No statistically significant differences were found with participants’ years of experience or profession type. Most participants, 43/62(69%), preferred the MAMI chart and reported it to be easier and faster to use than traditional tools. We conclude that weight-for-age assessment is quicker and more accurate using the newly-developed MAMI slide chart as opposed to traditional methods. It should be further field tested in other settings since the potential to improve the efficiency and effectiveness of treatment programmes is great.

## Introduction

Malnutrition is the “deficiency, excess or imbalance of energy, protein or other nutrients which adversely affects body function and/or clinical outcome” [1]. Infants and young children are the most vulnerable, with malnutrition underlying some 45% of all deaths in children aged under 5 years globally [2]. Long-term adverse effects are also now recognised. For example, survivors of malnutrition in childhood have a greater risk of non-communicable diseases such as heart disease and diabetes as adults [3]. They also risk not achieving their full developmental and cognitive potential [4]. For these reasons, preventing and treating malnutrition is a major global health priority and it is concerning when the latest Global Nutrition Report (GNR) highlights worsening global hunger [5].

Identifying malnourished and at-risk children is the essential first step to offering them effective treatment and support. Anthropometry, the measure of body size and shape, is widely used for this. Though an indirect and imperfect measure of malnutrition, anthropometry is practical, useful and is widely used in clinical and programmatic settings [6, 7]. Different anthropometric indicators have different advantages and disadvantages. None is a ‘gold standard’: what matters is how well they predict (i.e. how closely they are associated with) severe adverse outcomes, notably mortality [8, 9]. Prevalence of malnutrition varies according to indicator used. Some 22% of children aged under 5 years (U5) are stunted (low height-for-age) and 6.7% are wasted (low weight-for-age) [5]. Underweight (low weight-for-age) affects 12.6% of U5s globally but ranges widely: 0.8% in high income countries; 19.3% in low-income countries; 27.4% in South Asian countries [10].

Weight-for-age is currently used by many countries in Growth Monitoring Programmes (GMP) for younger infants and children [11]. GMPs aim to improve nutrition and health by identifying problems early. Recently, there is increasing in wider use of weight-for-age because:

- Reviews show that it is the best indictor of mortality risk in infants aged under 6 months (infants u6m) [12, 13]. It should thus be a priority indicator for enrolment to malnutrition treatment programmes for this age group [14].
- It could bridge gaps between GMPs and therapeutic/supplementary feeding programmes (TFPs, SFPs). Historically, the latter use wasting rather than underweight as their core admission criteria [7, 15]. This mismatch creates referral difficulties. However, recent data shows that children who are concurrently stunted as well as wasted have a particularly high risk of death [16, 17] and it turns out that such concurrence is reflected by low weight-for-age [18]. TFPs and SFPs might thus also benefit from knowing weight-for-age status.

Knowing if child’s weight-for-age is normal or low or very low is key to enrolling him or her into appropriate prevention or treatment programmes.

Weight is compared against an age and sex-matched reference group. Interpretation of weight-for-age is traditionally done using growth charts and look-up tables (**S1 Appendix** for examples of these). Whilst simple to use in theory, evidence shows many practical challenges which lead to inaccuracies and errors in practice (e.g. incorrect plotting; measurements not plotted at all; wrong interpretation of growth chart data) [19, 20]. Building on previous work with a weight-for-length ‘slide-chart’ [21, 22], we developed a novel weight-for-age assessment tool. We coined this the “MAMI chart” after our wider programme of work aiming to “Manage small, nutritionally At-risk Infants and their Mothers” [23]. Though widely applicable to young children, weight-for-age is particularly relevant for this group since it is likely to form the main future programme admission criteria [12]. The MAMI chart is a low-cost, appropriate technology [24] tool designed to simplify, speed and improve the accuracy of weight-for-age assessment.

The aim of this study was to evaluate the performance and acceptability of weight-for-age assessment using the new MAMI chart in comparison to the two traditional methods. We hypothesise that the MAMI chart will be both quicker and more accurate as compared to look-up tables and growth charts. Our objectives were to:

- Test if using the MAMI chart improves the accuracy and speed of nutritional assessment compared to look-up tables and growth charts.
- Evaluate user preferences for MAMI chart vs look-up tables and growth charts.

## Methods

### Study design

This was a prospective, cross-over diagnostic study evaluating a prototype version of the new MAMI chart against two traditional, long established weight-for-age assessment tools: growth charts and look-up tables. We followed the STARD checklist for diagnostic studies for reporting our work (**S2 Appendix**)

### Study participants and setting

Study participants were health and public health professionals working or studying in the UK. They included postgraduate MSc students at the London School of Hygiene & Tropical Medicine (LSHTM) who come from a variety of different professional backgrounds. They also included clinical staff working in neighbouring institutions who have training or experience in assessing children’s nutrition status. The core eligibility criterion was a general understanding of public health, child health, nutrition, and growth.

Participants were recruited as volunteers in response to advertisements posted: on the LSHTM electronic notice board; via internal email lists; via personal and institutional social media accounts; via the ENN (Emergency Nutrition Network) website, a public forum aimed at global health nutritionists (https://www.en-net.org/forum/19.aspx). They were enrolled on a first-come, first-serve basis using an online booking system to facilitate mutually convenient times.

Surveys were conducted in July and August 2022 in London, UK. All were conducted in-person at LSHTM or in nearby public spaces since a physical copy of the charts had to be used.

### Test methods

#### Index method—Development and use of the MAMI chart

**Fig 1:**
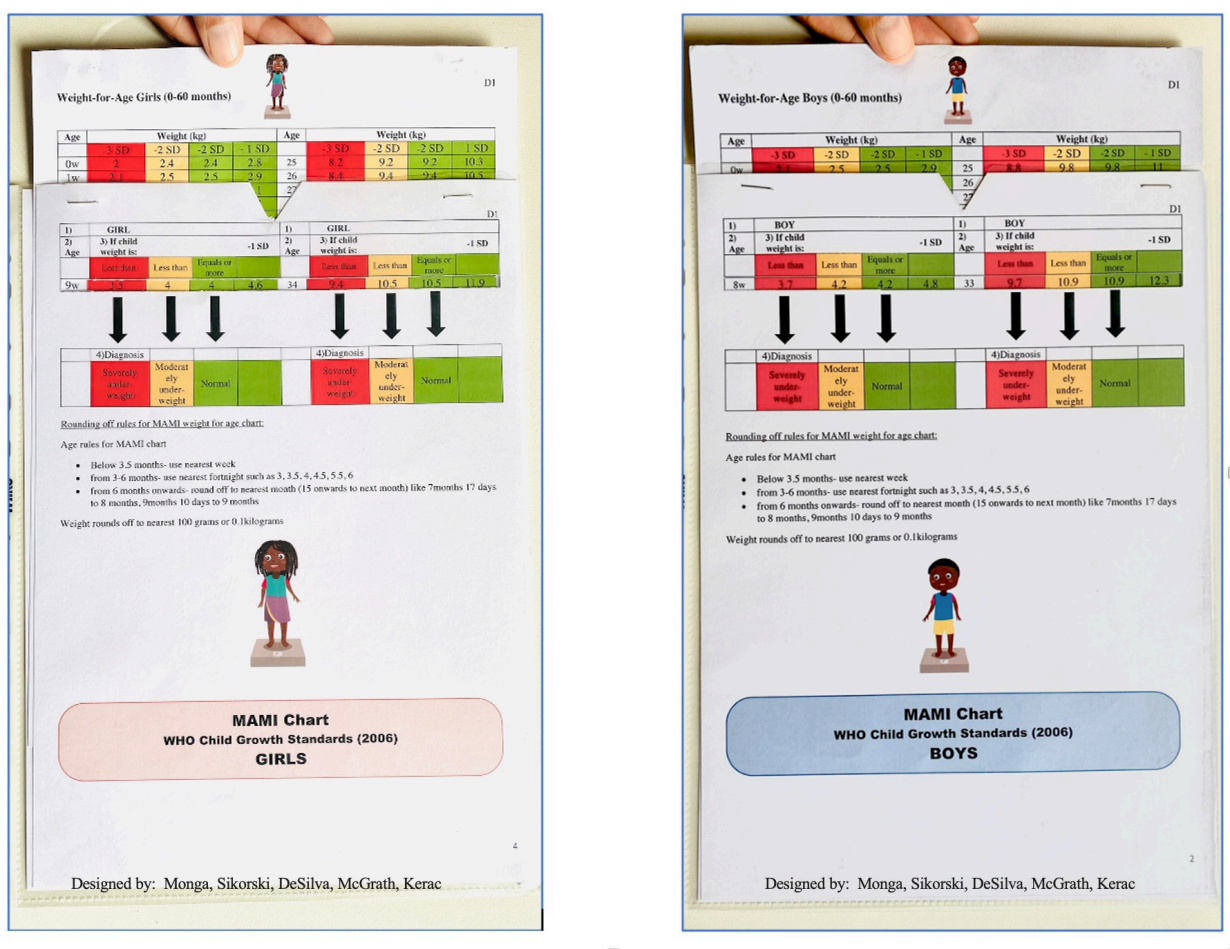
The MAMI chart front and reverse sides. (designed by: Monga, Sikorski, DeSilva, McGrath, Kerac).

The new MAMI chart (Fig 1) was inspired by a weight-for-length slide chart we previously developed [21]. The chart is designed for use in infants and children aged 0 to 60 months. It classifies a child’s nutritional status as severely underweight, moderately underweight or normal (not underweight) based on their weight-for-age Z-score (standard deviation score). The version we tested in this paper was chosen by consensus between the authors from three initial prototypes (**S3 Appendix).**

**Fig 2:**
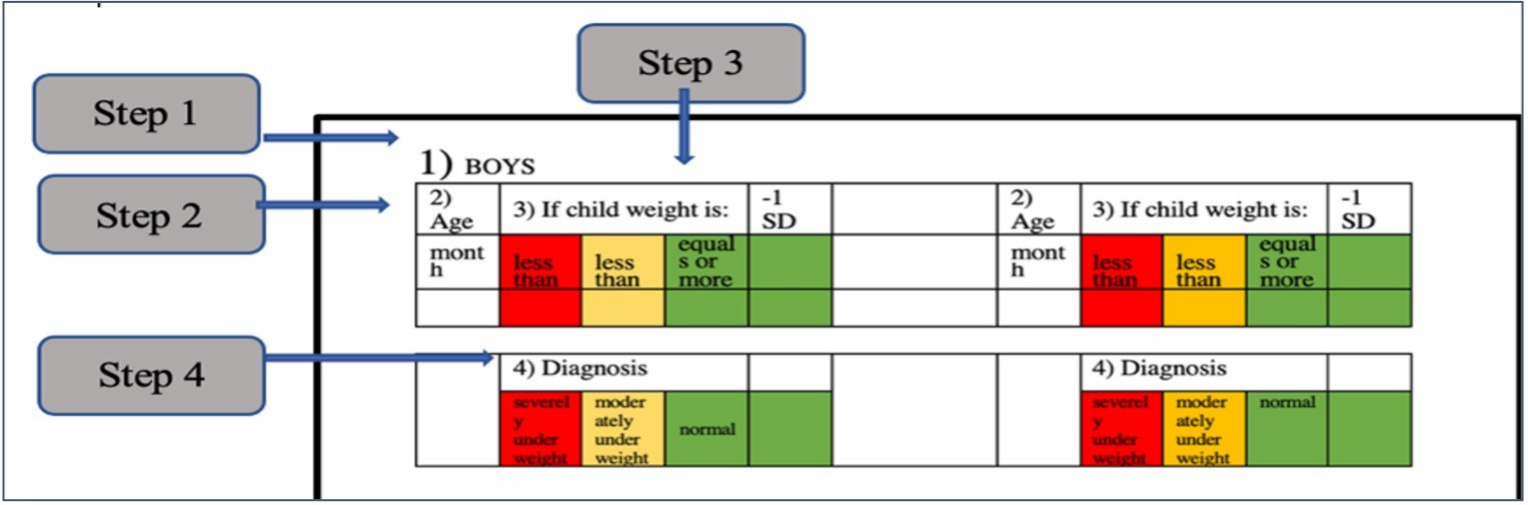
Steps to use MAMI weight-for-age slide chart.

Fig 2 summarises chart use and **S4 Appendix** gives further details on how it is used in practice. A user initially determines a child’s age and measures their weight. This is followed by step 1 of the chart: the user choses either the boy or girl side. In step 2, the chart insert is slid up or down so that the age is shown in the first ‘window’ on the left. In step 3, the user looks at weight figures in the corresponding windows to the right. These indicate what weight is normal; what is low (<-2 standard deviations, Z-scores, below the reference population median); what is very low (<-3 Z-scores below the reference population median) for that age. Finally, in step 4, the child’s actual weight is compared to these reference numbers to simply and rapidly classify the child as: normal, moderately underweight or severely underweight.

Weight, age and sex variables used for the chart are from the 2006 WHO growth standards (expanded tables for constructing national health cards) [25]. Age increments change according to three age bands: weekly increments from 0–13 weeks of life; fortnightly increments from ages 3–6 months; monthly increments from six to 59 months. These groups reflect growth patterns in early childhood: growth is the fastest in first three months of life; then it slows during ages three to six months; after six months it slows further still and becomes steady [26].

#### Other test methods

*Growth charts*. Weight-for-age growth charts show age in weeks or months on the y-axis and age on the x-axis. Continuous lines drawn on the chart show: weight-for-age z-score (WAZ) -2, as a red line; WAZ <-3, as a black line. If a child’s weight plot falls below the red line, he/she is underweight; if below the black line, he/she is severely underweight. We used standard WHO charts **(S1 Appendix)**.

*Lookup tables*. These present weight-for-age data in a table which is similar to the insert used by the MAMI chart. Users have to look up the child’s age on the left column of the chart and compare his/her actual weight against columns which show WAZ -2 and WAZ -3 respectively. Again, we used standard WHO tables (**S1 Appendix**).

### Reference standard

The ‘gold standard’ weight-for-age classification was based on the actual, cross-checked WAZ for a number of hypothetical scenarios with a given weight and age. Normal weight was defined as: WAZ ≥-2; moderate underweight was WAZ ≥-3 to <-2; severe underweight was WAZ <-3. These are standard cut-offs which have long been used in global child health/nutrition [6].

### Testing the MAMI chart vs growth charts and lookup tables

We developed 25 hypothetical clinical scenarios which presented a child’s sex, weight and age. The test task was to correctly classify as many of these as possible into normal weight, moderately underweight or severely underweight. Numbers in each category were roughly balanced. Eight of the 25 scenarios had the WAZ score exactly at -2 or -3 z score (borderline scenarios) to make them more challenging. The answer key was developed for the questionnaires and were cross-checked for any errors, prior to the evaluation.

Two questionnaires were used:

- a general questionnaire containing the 25 scenarios which asked participants to select what they determined to be the correct anthropometric classification in each case (normal, moderately underweight, severely underweight) (**S5 Appendix**). Basic participant demographics were also collected by this questionnaire.
- an acceptability questionnaire (**S6 Appendix)** which asked for feedback on the three different assessment tools/methods.

Questionnaires were administered electronically via Microsoft forms (Office 365, Microsoft, USA). To optimise questionnaire validity, they were: based on those used in a similar previous project [22]; reviewed and updated prior to this project; pilot tested before use with participants.

On the day of the test, after going through the participant information sheet and obtaining written informed consent, each participant was given a basic orientation about how to use all three test methods: the new MAMI slide chart, look-up tables, and growth charts.

Each participant was then given four minutes, timed using a stopwatch, to complete as many of the 25 scenarios in the first questionnaire as possible. This was repeated three times so that each participant used each of the three tools (MAMI slide chart, look-up tables, and growth charts). Tool order was randomised each time.

The 4-minute time limit for a maximum of 25 scenarios was deliberately difficult so as to mimic the time and stress pressures of real-life scenarios in busy clinical settings.

Finally, each participant also completed the acceptability questionnaire (**S6 Appendix**) to understand their preferences and gather ideas of how to improve future versions of the MAMI tool. This questionnaire consisted of 15 questions, of which 12 answers were on a Likert scale, two were simple one-choice questions, and one was open-ended for suggestions to improve the MAMI chart.

Correct answers to the different scenarios were not shared at the end of the study and neither were participants informed of their final scores. This was because we were not testing the skills and abilities of our participants, but rather the relative performance of the three different methods of assessing underweight status.

### Sample size

We based our sample size calculations on a similar previous study comparing a weight-for-length slide chart vs look-up tables [22]. In that study, a difference in diagnostic accuracy of 7 percentage points was observed, with mean accuracy of 83% for the new slide chart (SD 22) and 76% for (SD 19.5). Assuming similar results in our study would have required 139 participants per group (power 80%, 95% CI), using a sample size method for comparing two means (OpenEpi, https://www.openepi.com/SampleSize/SSMean.htm). We were however logistically constrained by available time to perform our work so aimed for a minimum of 50 participants per group. With power 80% and 95% CI, 50 per group would be sufficient to observe a minimum 12 percentage points difference in mean accuracy (SD 21 per group). 70 per group would give enough power to observe 10 percentage points difference (SD 21 per group)

Through our cross-over design, participants each acted as their own control, with all using all three methods to calculate and interpret WAZ categories.

### Exposure variable and confounders

Each weight-for-age nutritional assessment tool (the MAMI chart, look-up tables, growth charts) was the exposure variable. We also considered key confounders. Years of experience in nutrition or public health were divided into four subgroups: less than 5 years, 5–10 years, 10–15 years and over 15 years. In the acceptability questionnaire we also asked about any direct experience with any of the three methods. Background profession was divided into five subgroups: students, researchers, doctors, nurses, midwives, and others. These broad categories also helped us maintain anonymity. Participants’ gender was also recorded.

### Outcome variables

#### Primary outcomes

Co-primary outcomes were diagnostic accuracy and response rate. Diagnostic accuracy was assessed by calculating each tool’s percentage of correct responses to clinical scenarios attempted in total. The response rate with each tool was calculated by summing the average number of scenarios attempted by each participant in 4 minutes (the maximum scenarios possible were 25 per tool in 4 minutes).

#### Secondary outcomes

Secondary outcomes were perceived ease of use and user preferences for each tool. These were measured by the percentage of participants expressing a preference for each of the three tools. We also reported responses to the Likert-scale questions using simple descriptive analysis. Finally, we summarised free-text suggestions to improve future versions of the MAMI chart.

#### Potential confounders

We assessed two major potential confounders. One was prior experience background professional training. The other was years of experience in public health/nutrition.

#### Bias

It was not possible to blind the participants as to which nutritional assessment tool they were using. Hence, they were used in random order. The order of the clinical scenarios in the first questionnaire was also randomly varied to reduce any learning bias when repeated with the subsequent tools.

#### Statistical analysis

After completing the survey, data was downloaded from MS Forms in Excel format and from there transferred to Stata version 17.0 (28) for analysis.

Diagnostic accuracy and response rate with each tool were compared using the chi-square method and ANOVA. A p-value of <0.05 was taken to be statistically significant.

Finally, multiple logistic regression analysis was done to explore any statistically-significant differences in diagnostic accuracy.

#### Ethical considerations

Ethical approval was obtained from the London School of Hygiene and Tropical Medicine MSc ethics committee (reference 27239). Informed written consent was taken from each participant, and a participant information sheet was provided to each participant, sharing details about the study

## Results

**Fig 3:**
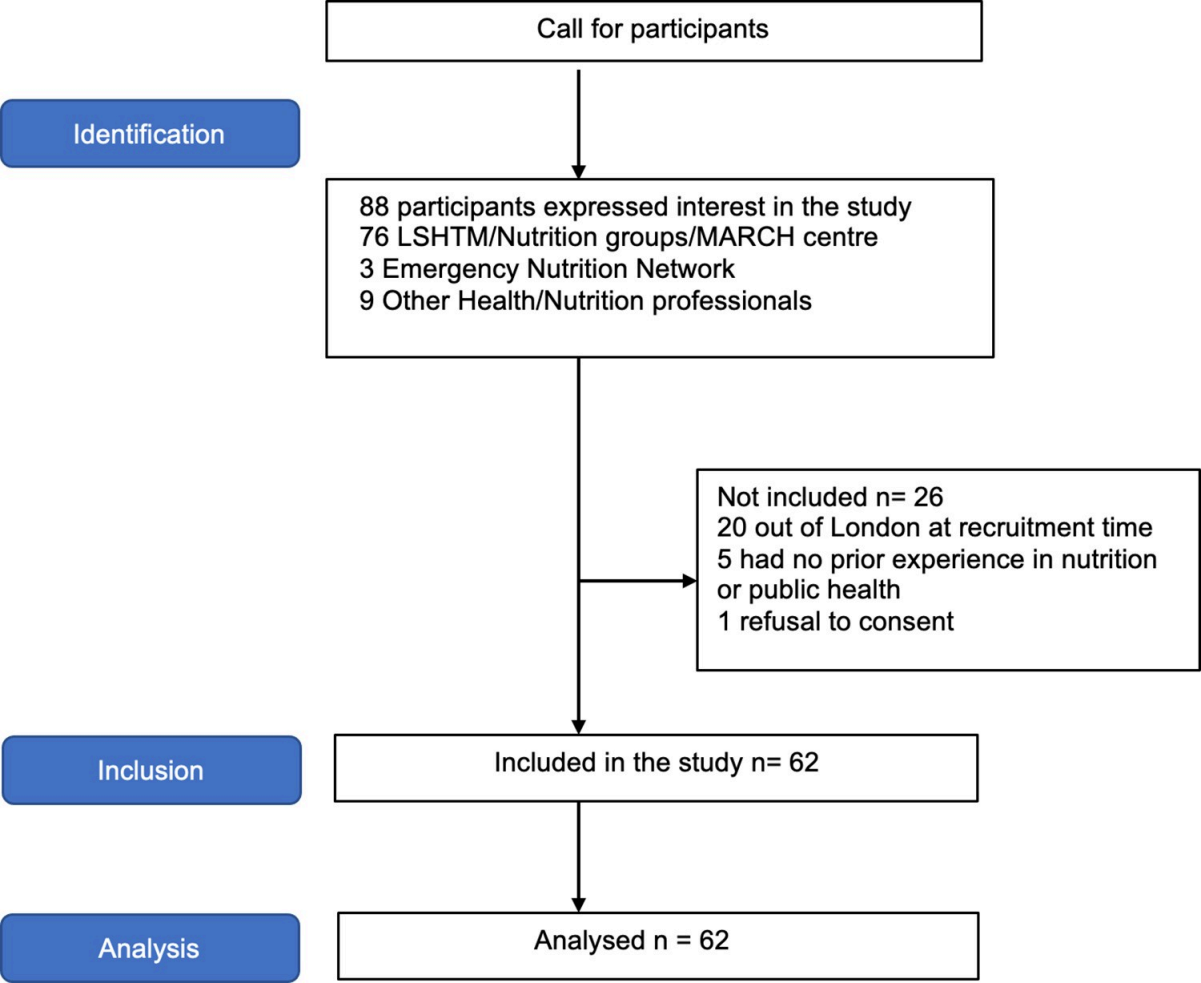
Study participants flow diagram.

A total of 88 participants approached after the invitation, out of which 62 were enrolled. Reasons for exclusion were: 20 were based out of London during the research period and unable to attend the in-person assessment, 5 had no relevant prior experience in nutrition or public health; one declined consent after learning more about the study **(**Fig 3).

### Demographic characteristics of participants

A total of 62 participants were included in the final analysis. Of these, 44 (71%) were female, and 18 (29%) were male. Forty-eight (77%) participants had 0–5 years of prior experience in nutrition or public health. As for profession, 28 (45%) of all participants were students, 24 (39%) were doctors, 7 (11%) were from other professions, 2 (3%) were nurses, and 1 (2%) was a researcher. There was no missing data for basic demographic variables. Table 1 shows the basic demographic characteristics of the participants.

**Table 1:** Basic demographic characteristics of study participants.

	N (62)	%
**Gender**		
Male	18	29%
Female	44	71%
**Years of experience**		
0 to 5 years of experience	48	77%
5 to 10 years of experience	5	8%
10 to 15 years of experience	7	11%
over 15 years of experience	2	3%
**Profession** *		
Student	28	45%
Researcher	1	2%
Doctor	24	39%
Nurse	2	3%
Other professions	7	11%

*There were no participants who were midwives by profession

### Primary outcomes

From the hypothetical clinical scenarios in the questionnaire, a total of 353 responses were recorded for all participants when using growth charts, 438 responses using look-up tables and 445 responses using the MAMI slide chart. The maximum possible number of responses would have been 25*62 = 1550.

Table 2 summarises the number of responses and distribution by each tool.

**Table 2:** Total number of responses and distribution by each tool.

	Growth charts (n = 62)	Look-up tables (n = 62)	MAMI chart (n = 62)
**Total number of responses** (max possible = 25*62 = 1550)	353	438	445
**Total number of correct responses**	217	308	351
**Diagnostic accuracy of tool** (correct responses / total responses)	61%	70%	79%
**Average number of scenarios (± SD) attempted per participant in 4 minutes**	5.71 ± 2.08	7.06 ± 2.61	7.18 ± 2.89
**Average number of correct responses (± SD) per participant in 4 minutes**	3.5 ± 2.19	4.97± 2.50	5.66 ± 2.69
**Average number of correct responses per participant in projected to 60 minutes**	53	75	85

Diagnostic accuracy was highest for the MAMI chart with 79% assessments correctly made (351/445). This was followed by the look-up tables at 70% (308/438) and was lowest for the growth charts at 61% (217/353). A chi-square test showed a statistically significant difference between the three methods (p-value< 0.01). A statistically significant difference was also found when comparing each tool independently with the other (chi-square method, growth charts vs look-up tables p-value <0.01, look-up tables vs MAMI chart p-value <0.01, growth charts vs MAMI chart p-value <0.01).

The total number of responses in the given time (speed of use of the tool) was compared using the chi-square method. Statistically significant differences were found between the three groups (p-value <0.01). The average number of scenarios attempted in 4 minutes by each tool is also shown in Table 2: there was again a statistically significant difference between the three tools (one-way ANOVA of repeated measures, p-value <0.01).

Also in Table 2 is the average number of scenarios identified correctly by each tool: there was a statistically significant difference between the three (one-way ANOVA, p-value <0.01).

Extrapolating results from Table 2 into patients who could theoretically be assessed and correctly classified in an hour is approximately 85 by the MAMI chart compared to 75 using the look-up tables and 53 by growth charts.

Table 3 explores the borderline scenarios in detail since these may be more prone to user errors than scenarios where a child is well above or well below the -2 and -3 WAZ threshold values. The MAMI chart had a higher diagnostic accuracy for both borderline and other cases. In contrast, growth charts and look-up tables showed poor diagnostic accuracy for borderline cases. Inter-chart differences were statistically significant for both borderline scenarios (chi-square method, p-value <0.01) and for non-borderline ones (chi-square method, p-value <0.01).

**Table 3:** Distribution of responses in borderline scenarios by each tool.

	Growth charts (n = 62)		Look-up tables (n = 62)		MAMI chart (n = 62)
	Borderline scenarios	Other	Borderline scenarios	Other	Borderline scenarios
**Total number of responses**	96	258	131	307	124
**Total number of correct responses**	46	171	58	250	81
**Diagnostic accuracy of tool**	48%	66%	44%	81%	65%

The total number of responses and distribution of responses by profession are shown in Table 4. There were no statistically significant differences found between participant’s profession type in diagnostic accuracy (chi-square method, p-value 0.85), the average number of scenarios attempted in 4 minutes (one-way ANOVA, p-value 0.13) and the average number of correct responses in 4 minutes (one-way ANOVA, p-value 0.67).

**Table 4:** Total number of responses and distribution of responses by profession.

Profession	Student (n = 28)	Doctor (n = 24)	Others*(n = 10)
**Total number of responses**	523	508	206
**Total number of correct responses**	381	354	141
**Diagnostic accuracy**	73%	70%	68%
**Maximum number of responses possible (25 scenarios per participant per tool)**	25*3*28 = 2100	25*3*24 = 1800	25*3*10 = 750
**Average number of scenarios attempted (± SD) per participant in 4 minutes**	6.23 ± 2.14	7.06 ± 2.99	6.87 ± 2.84
**Average number of correct responses (± SD) per participant in 4 minutes**	4.54 ± 2.09	4.92 ± 3.02	4.7 ± 2.94
**Average number of correct responses per participant in projected to 60 minutes**	68	74	71

The total number of responses and distribution of responses by years of experience are shown in Table 5. There were no statistically significant differences found between participants’ years of experience in diagnostic accuracy (chi-square method, p-value 0.75), the average number of scenarios attempted in 4 minutes (one-way ANOVA, p-value 0.63) and the average number of correct responses in 4 minutes (one-way ANOVA, p-value 0.85).

**Table 5:** Total number of responses and distribution of responses by years of experience.

Years of experience	0–5 years (n = 48)	over 5 years*(n = 14)
**Total number of responses**	965	272
**Total number of correct responses**	681	195
**Diagnostic accuracy**	71%	72%
**Total number of responses available (25 scenarios per participant per tool)**	25*3*48 = 3600	25*3*14 = 1050
**Average number of scenarios attempted (± SD) per participant in 4 minutes**	6.70 ± 2.52	6.48 ± 2.97
**Average number of correct responses (± SD) per participant in 4 minutes**	4.73 ± 2.45	4.64 ± 3.15
**Average number of correct responses per participant in projected to 60 minutes**	71	70

*Over 5 years of experience included 5–10 years, 10–15 years and over 15 years of experience

Statistically significant difference was present for diagnostic accuracy by chart type using multiple logistic regression analysis p-value <0.01, but no statistical difference was found in accuracy by gender, type of profession groups and years of experience groups.

### Secondary outcomes

#### Ease-of-use

45 (73%) of participants found the MAMI slide chart ’slightly easier’ or ’much easier’ as compared to the look-up tables (fisher exact test p-value <0.01). Similarly, 56 (90%) participants found the MAMI slide chart ’slightly easier’ or ’much easier’ as compared to growth charts (fisher exact test p-value <0.01). Fifty-one (82%) found the MAMI chart ’slightly faster’ or ’much faster’ as compared to traditional tools (fisher exact test p-value <0.01).

#### When asked how stressful using the tools was

52 (84%) and 47 (76%) of 62 participants found growth charts and look-up tables, respectively, either ’slightly stressful’, ’moderately stressful’ or ’highly stressful’. In contrast, only 22 (35%) participants found the MAMI slide chart as ’slightly stressful’, and 40 (65%) participants indicated it as ’not stressful’.

#### Prior experience of using tools

16 (26%) of participants have never used a tool like a look-up table before. Similarly, 7 (11%) have never used a growth chart, and 48 (77%) have never used a tool like the MAMI slide chart.

#### Preference

43 (69%) participants indicated that they would prefer to use the MAMI slide chart when working in clinical settings. Only 10 (16%) would prefer to use a look-up table, and 9 (15%) would like to use the growth charts (fisher-exact test, p-value<0.01)

#### Design preference

The three original MAMI chart prototypes (**S3 Appendix**) were also shown to the participants, and they were asked about their preferences. Thirty-four (55%) participants preferred design I as was ultimately chosen for full testing (boy and girl z scores are on either side of the slide chart), 4 (6%) preferred design II (boys and girls z scores are on completely different charts) and 24 (39%) preferred design III (boy and girl z scores are on side-by-side-parallel arrangement).

#### Suggestions for improvement

Most participants appreciated the design and concept of the new tool. Common suggestions were to use contrasting colours for boys and girls, to have an easier sliding mechanism for charts, to simplify rounding rules for age, and to mention units for age clearly.

## Discussion

### Main findings

As used by a diverse group of health and public health students and professionals, we found that the new MAMI chart was superior to traditional lookup tables and growth chart in determining underweight status from raw weight and age values. It was significantly (statistically) more accurate at classifying a child as normal/moderately underweight/severely underweight. Accuracies of 79%, 70% and 61% for the MAMI chart, look-up tables and growth charts respectively are also clinically significant. Extrapolated to clinical practice, these differences could mean that large numbers of affected infants might not get access to the treatments they need if the latter two traditional assessment methods are used. The superior performance of the MAMI chart cannot be explained by borderline cases around WAZ <-2 and <-3 alone. These are most difficult to classify correctly yet the superior performance was also observed in non-borderline cases. Neither can it be explained by prior, pre-study familiarity with the tools. On the contrary, these strikingly positive results followed a very rapid learning curve whereby few participants had ever before used a slide-chart tool like the MAMI chart whereas many had used look-up tables and most had experience of growth charts. Years of professional work experience and professional background also made no difference to the success of the MAMI chart over the two traditional methods.

The new MAMI chart was also significantly quicker to use. Again, statistically significant differences are also likely to be clinically meaningful: 85 cases could be assessed in an hour using the MAMI chart but only 75 and 53 using the look-up tables and growth charts.

Finally, the new MAMI chart was strongly preferred in user feedback: this acceptability is a key feature for any new screening/testing strategy.

Reasons for these positive results need to be understood. Whilst the superior performance of the MAMI chart is unlikely due to familiarity and prior experience, it is possible that a novelty factor played a role. Maybe users payed more attention and took greater care using a novel and thus more interesting and engaging method. We cannot exclude this possibility, but we can say that the MAMI chart was quick: at least any novelty factor still produced the quickest assessment of all three methods.

Given that blinding was impossible, we also cannot exclude a possibility of bias in how the three methods were explained and presented to study participants. Again, however this is unlikely. The fact that many more users were already familiar with growth charts and lookup tables should have led to those being better superior given that users effectively had more pre-study information /experience / explanation of those two traditional methods.

Tool simplicity may play a role. Having few steps all clearly laid out and described likely helps make the MAMI chart quicker to use and contributes to its superior diagnostic accuracy. In contrast, using growth charts can take time with multiple steps required (e.g. looking up the correct x axis value; looking up the correct y axis value; correctly plotting a point where they intersect; comparing that point to the -2 and -3 z-score lines). Errors can easily occur at each of those steps: the more steps, the more potential for error overall. Look-up tables are not dissimilar to the MAMI chart but might take longer and be less accurate because there are no guides or reminder on their use printed on the tables themselves. Look-up table errors are also possible when reading along rows and accidentally focusing on values above or below the correct row. Such errors are eliminated by the MAMI chart since the ‘window’ system means that only one row can be highlighted at a time. Lastly, clear pictures of a boy or girl over the front sheets of the MAMI chart reduces errors in using the girls’ table when measuring a boy or vice versa.

### Sub-group findings

In our study, accuracy and the average number of scenarios correctly classified were similar for users of different professional backgrounds and different durations of professional experience. Differences in these subgroups is plausible so their absence is notable. The lack thereof may highlight the benefits and superiority of the MAMI chart for a wide range of users. We also though acknowledge small sample size for subgroups. Also, our questions were broad in nature so might not have captured any subtler differences (e.g. one year’s experience as a health visitor plotting growth charts on a near daily basis is far more relevant experience than 30 years as an adult physician who would not have plotted a growth chart since medical school)

### Secondary outcomes

Most participants, 69%, preferred the MAMI slide chart over traditional tools, despite most, 77% using it for the first time. They felt that the MAMI charts were faster, less stressful and required fewer corrections. This could be due to the MAMI slide chart’s overall design: a simple, slick format with a single chart including both boys and girls and all ages 0–60 months; a clearly described step-by-step process; precise interpretation of nutritional categories using colours and the innovative slide mechanism. We also acknowledge possible responder bias which readers should be mindful of when interpreting this data. Those volunteering for the study may be more predisposed to and interested in novel technologies and approaches. Hence possibly also more likely to like any new tool or gadget irrespective of design advantages/disadvantages.

### Research in context

As previously described, the MAMI weight-for-age slide chart used in this study was inspired by our previous MOYO weight-for-length slide chart which was designed to help with assessing wasted/acutely malnourished children [21]. MAMI slide chart test performance aligns with similar performance testing of the MOYO slide chart by Sikorski et al. in 2010 [22]. This worked with 61 medical students in Ethiopia and also found that a slide chart method was superior to standard look-up tables, with a mean accuracy of 83.2% vs 76.1%. The MOYO chart also reduced time needed for each correct diagnosis, and also had high user preference (78%). Taken together, these results show consistent advantages to slide-chart methods.

Despite a widespread presence in many national and international policies [11], our results are also consistent with other research which also shows problems with current ways of determining underweight status, especially when using growth charts. These are challenging because they involve multiple steps, each of which can introduce errors, leading to final misclassification. A 1991 study in Lesotho highlighted challenges in training healthcare workers to use growth charts [27]. In 2013, Mutoro et al. found that healthcare workers in Kenya had poor plotting accuracy and poor use of serial measurements to diagnose undernutrition in children [28]. A similar study by Wright et al. in 2012 among UK healthcare workers also showed poor plotting accuracy, with 64% of respondents making at least one major mistake [29]. More recently, a 2019 study from Ghana showed poor healthcare worker use of growth charts [30].

### Strengths and limitations

Our study’s main strength is its design: a randomised cross-over study whereby each participant acted as their own control. This limited many potential confounders. We presented our hypothetical clinical scenarios in random order, thus preventing possible learning bias when repeating the scenarios with different tools. Also, to prevent any learning-related biases, the three tools were presented in random order to each participant. Another strength was the diversity of the study participants. These ranged from doctors to nurses to MSc students in various public-health related fields. Their prior clinical and public health experience ensured that responses and feedback has value. Finally, our use of electronic data capture makes the study quick and easy and also reduced transcription errors as can happen when entering data from paper data collection forms.

Limitations include a relatively small sample size. This was not as much of a limit as it could have been since inter-chart diagnostic accuracy difference was greater than the 5% we’d originally anticipated. Hence final power was in fact adequate for the main inter-chart comparisons even if not sufficient for robust subgroup analysis.

Second, since ours was a cross-sectional study, blinding was impossible for both participants and for the assessor.

Third, our study design meant that the study was conducted in person with London-based participants. Not everyone who expressed interest could be enrolled. Neither could we directly work with many of the intended future users of the MAMI chart. These include community and clinic-level healthcare workers in low and middle income countries and in humanitarian settings. Future work needs to test MAMI chart performance with these directly–though we hypothesise that if MSc-level healthcare workers and students struggled with the two traditional methods, this will be all the more so with other less highly trained staff.

Moreover, the study was conducted in a quiet and distraction-free environment as opposed to a community or clinical setting with a high volume of cases and multiple other responsibilities. We also only focused on one aspect of overall health and nutrition assessment. In practice, an infant or child would also need to be clinically assessed, their age correctly determined and their weight correctly taken. These all take time and could impact final results even if those prior steps are exactly the same for all three final methods to interpret the obtained age and weight values. Future work could test tool performance in actual clinical scenarios rather than just hypothetical ones. In clinical work, far more other information is available to support final decisions about nutritional status and possible treatment. In our study, no other such parameters such as medical history, current and past dietary intake, anthropometric indicators, or clues were provided to the participants, which may or may not influence the participant’s decision regarding the nutritional assessment.

Finally, user preferences results of tools might be influenced by self-selection bias, as participants who are interested in novel things or gadgets are most likely to participate in this study.

### Implications for practice

Given resurgent interest in low weight-for-age as an admission criterion for treatment programmes for small and nutritionally at-risk infants aged u6m [12] and for identifying concurrently wasted/stunted older children in therapeutic and supplementary feeding programmes [16], our paper is timely. Separate work is of course needed to address the challenge of how best to determine accurate weight and accurate age, but we do seem to have an effective and acceptable solution to accurately determining whether weight-for-age is low or not based on these.

Potential for widespread use of our MAMI weight-for-age chart is highlighted by previous experience with our MOYO weight-for-length slide chart. In 2010 we obtained a small amount of funding to print and distribute this via health charity TALC (Teaching Aids at Low Cost), since taken over by publisher Healthbooks International (https://healthbooksinternational.org) In some 7 years after this initial launch, with no direct marketing, some 15,000 copies of the MOYO chart were sold. If each chart was used just once a working day to assess one child, in 7 years this means 7*15,000*260 = 27 million assessments made and facilitated. This could amount to a great amount of clinical time saved, children correctly assessed and enrolled into programmes (or not) and errors avoided. We hope for similar future success and impact for the MAMI chart.

We acknowledge competing interest from app-based electronic tools which might have a similar role and output as our MAMI chart. These however have their own challenges and problems [31]. Even free tools need ongoing maintenance and support and continuous updated to ensure they still work as background operating systems like Android are updated. Hence, we believe there is a continued and important role for paper- based tools like the MAMI chart. Evidence of this includes other paper based tools in widespread use such as pregnancy wheels, child development wheels, BMI calculator discs, and neonatal bilirubin wheel, among others. In contrast to electronic tools, paper-based tools are relatively robust, long-lasting and economical.

Future research should test improved versions of our MAMI chart as well as testing the approach in different settings so as to formally explore generalizability of our results to different healthcare worker populations in different settings. The prototype we tested in this paper was a hand-assembled version–future printed versions should also be tested before scale up. Formatting details such as clarity of instructions on the front and rear page might further improve the diagnostic accuracy, ease of use and speed of use of the tool.

Perhaps most concerning in our results was the poor performance of traditional tools such as growth charts and look-up tables. Whatever happens with the MAMI chart, these will also remain relevant but there needs for example to be more attention to pre-service and in-service training to best use these. Certainly, the growth chart will always be a useful and important way to assess linear growth–the MAMI tool alone cannot replace this roles.

Finally, more work is needed on the utility of weight-for-age in general. Whilst some reviews show its superior performance in identifying infants at high risk of death, the indicator only work if age can be accurately determined and weight can be accurately measured. This may not be the case in all settings, though DOB is far more widely known and documented in many countries today than even 5–10 years ago.

## Conclusions

Our results indicate that weight-for-age assessment is quicker and more accurate using our newly-developed MAMI slide chart as opposed to traditional methods. Accuracy and performance of growth charts and look-up tables is surprisingly poor. Given they are in widespread use in policy and programmes in many countries, improved training and supervision on these is urgent. Whilst the MAMI chart is a highly promising future development, it needs to be further field tested in other settings with other healthcare workers who will directly use it in future. Its’ potential to improve the efficiency and effectiveness of treatment programmes is great.

## Supporting information

S1 Appendix Growth charts, look-up tables. (DOCX)

S2 Appendix STARD-2015-Checklist_MAMI chart_v2.0. (DOCX)

S3 Appendix MAMI chart prototypes. (DOCX)

S4 Appendix MAMI chart use—detailed instructions. (DOCX)

S5 Appendix Main questionnaire—WAZ classification. (DOCX)

S6 Appendix Acceptability questionnaire. (DOC)
