# Supplementary material for: Identifying underweight in infants and children using growth charts, lookup tables and a novel “MAMI” slide chart: A cross-over diagnostic and acceptability study
Source: PLOS Glob Public Health. 2023 Aug 30;3(8):e0002303. doi: 10.1371/journal.pgph.0002303 (PMC10468082; doi:10.1371/journal.pgph.0002303)

S1 – Weight-for-age growth charts and look-up tables

1. WHO weight-for-age growth chart for girls' birth to 6 months (z-scores)


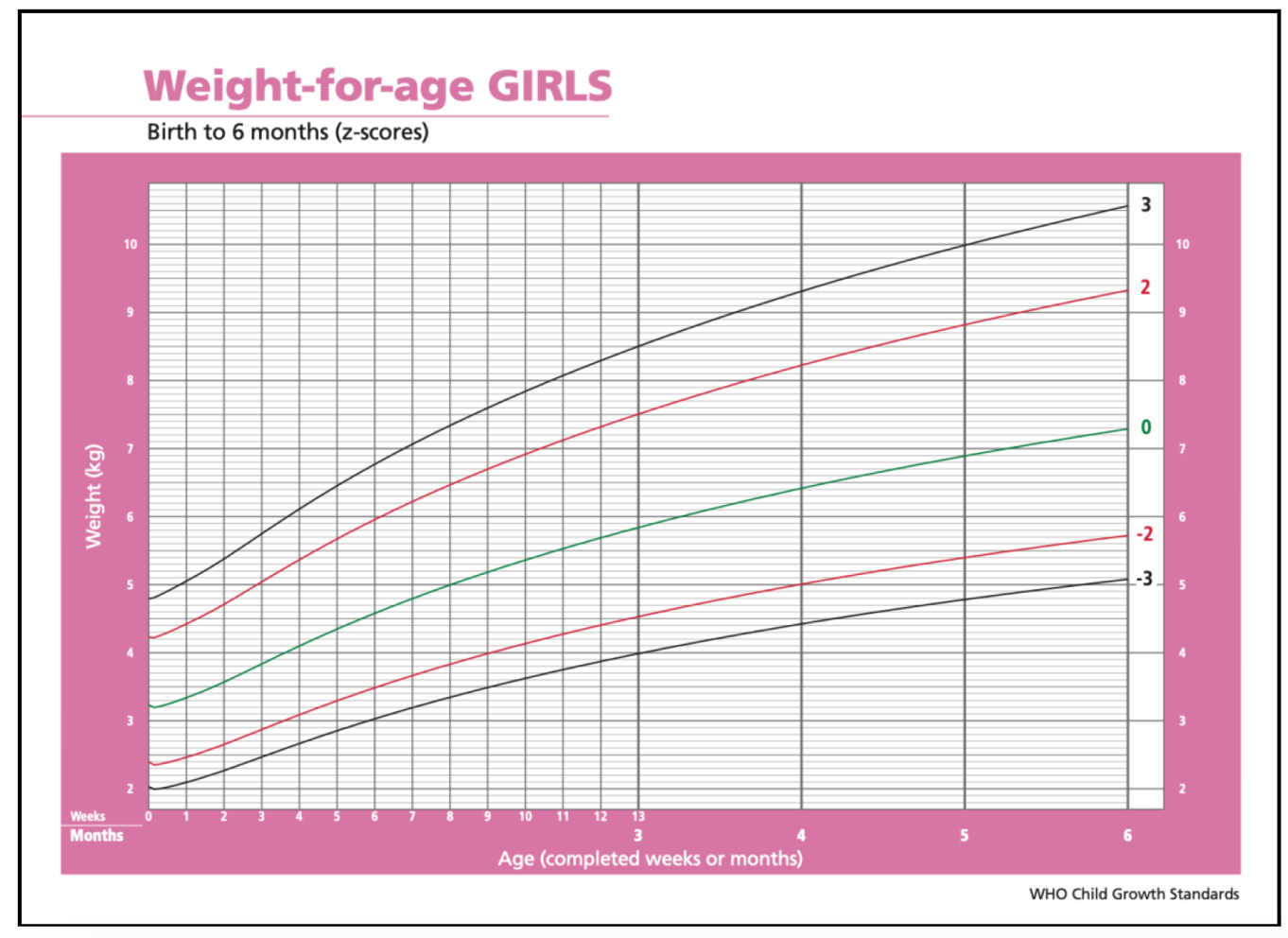


1. WHO weight-for-age growth chart for boys' birth to 6 months (z-scores)


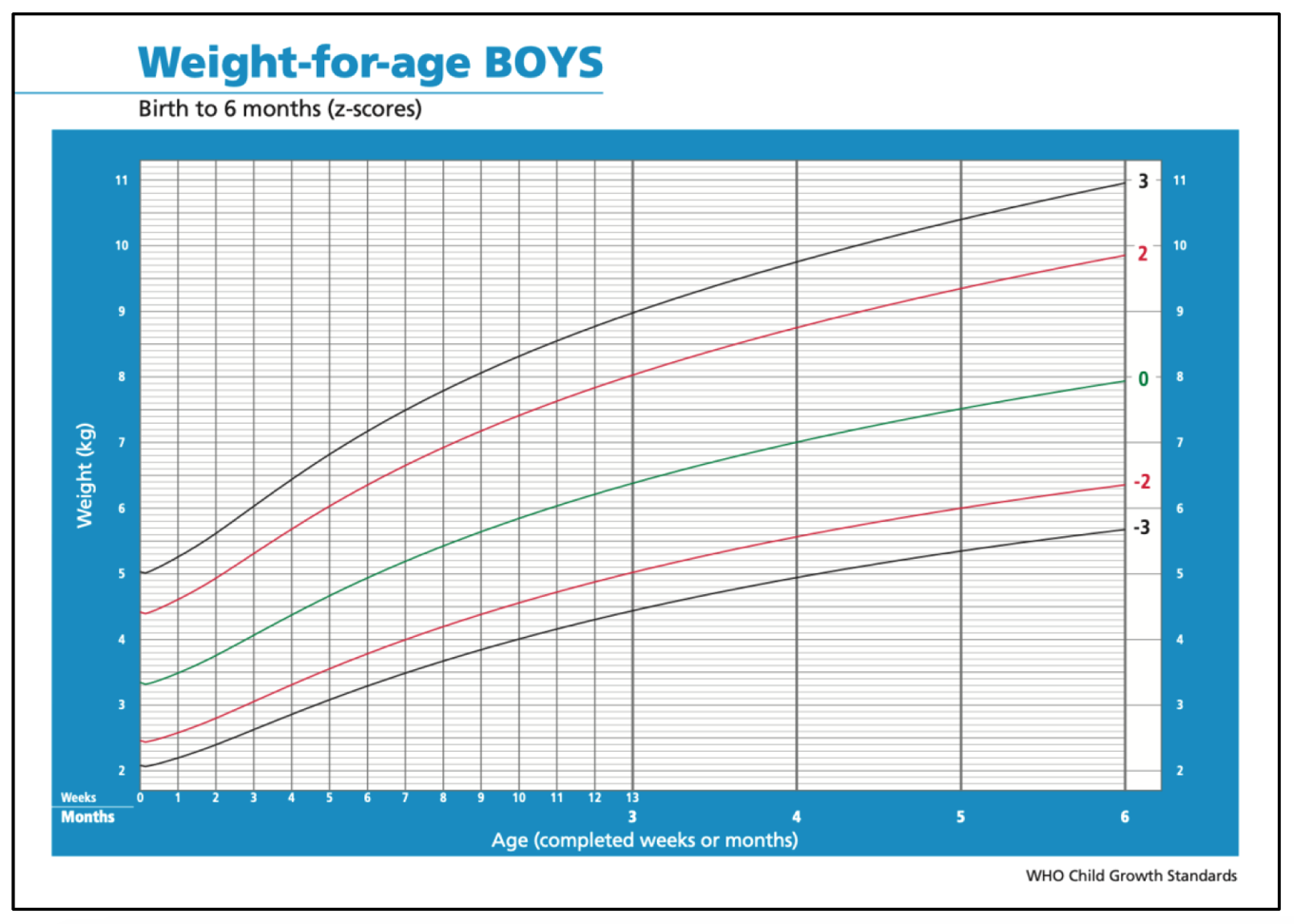


1. WHO weight-for-age growth chart for girls' birth to 5 years (z-scores)


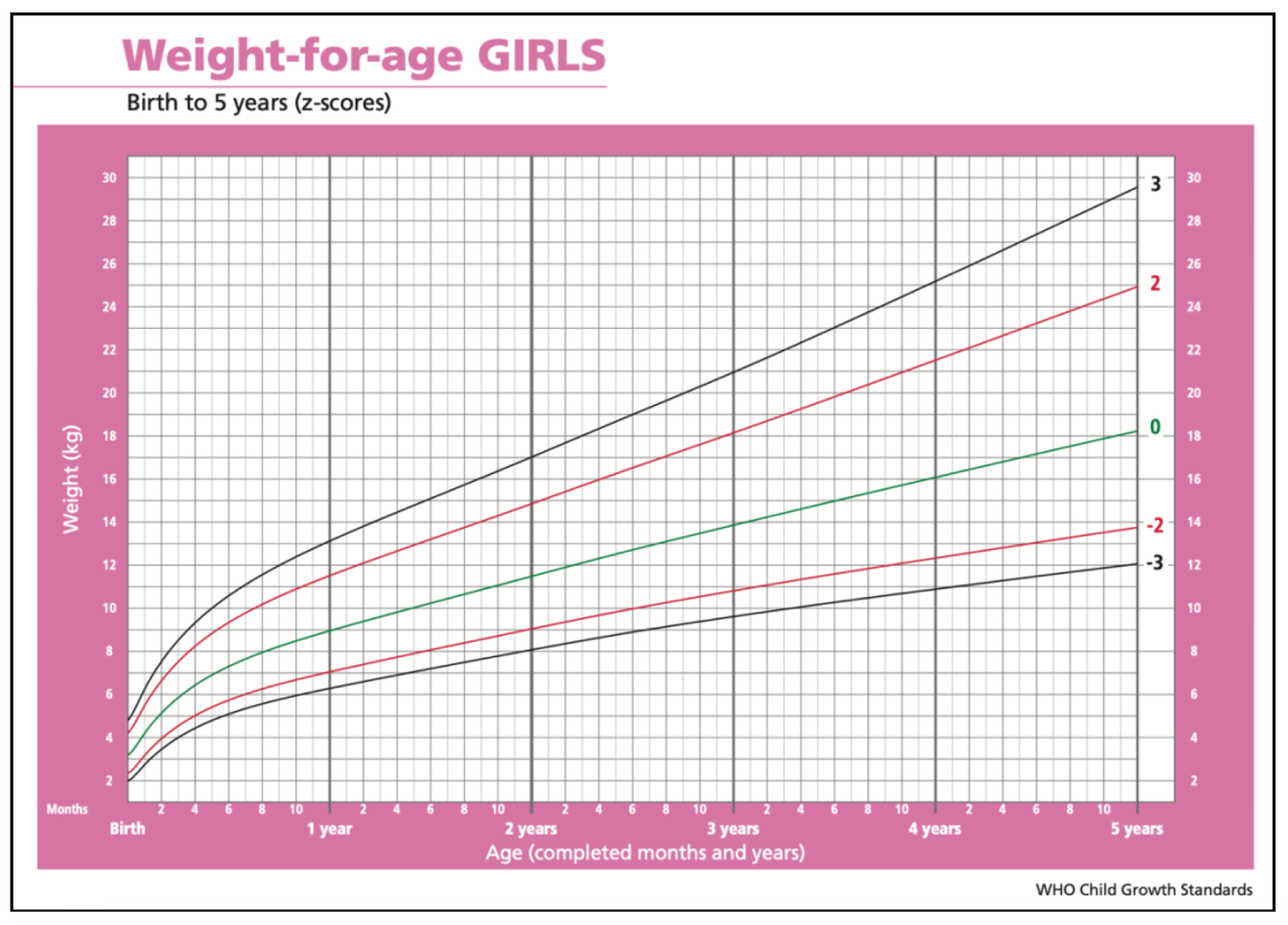


1. WHO weight-for-age growth chart for boys' birth to 5 years (z-scores)


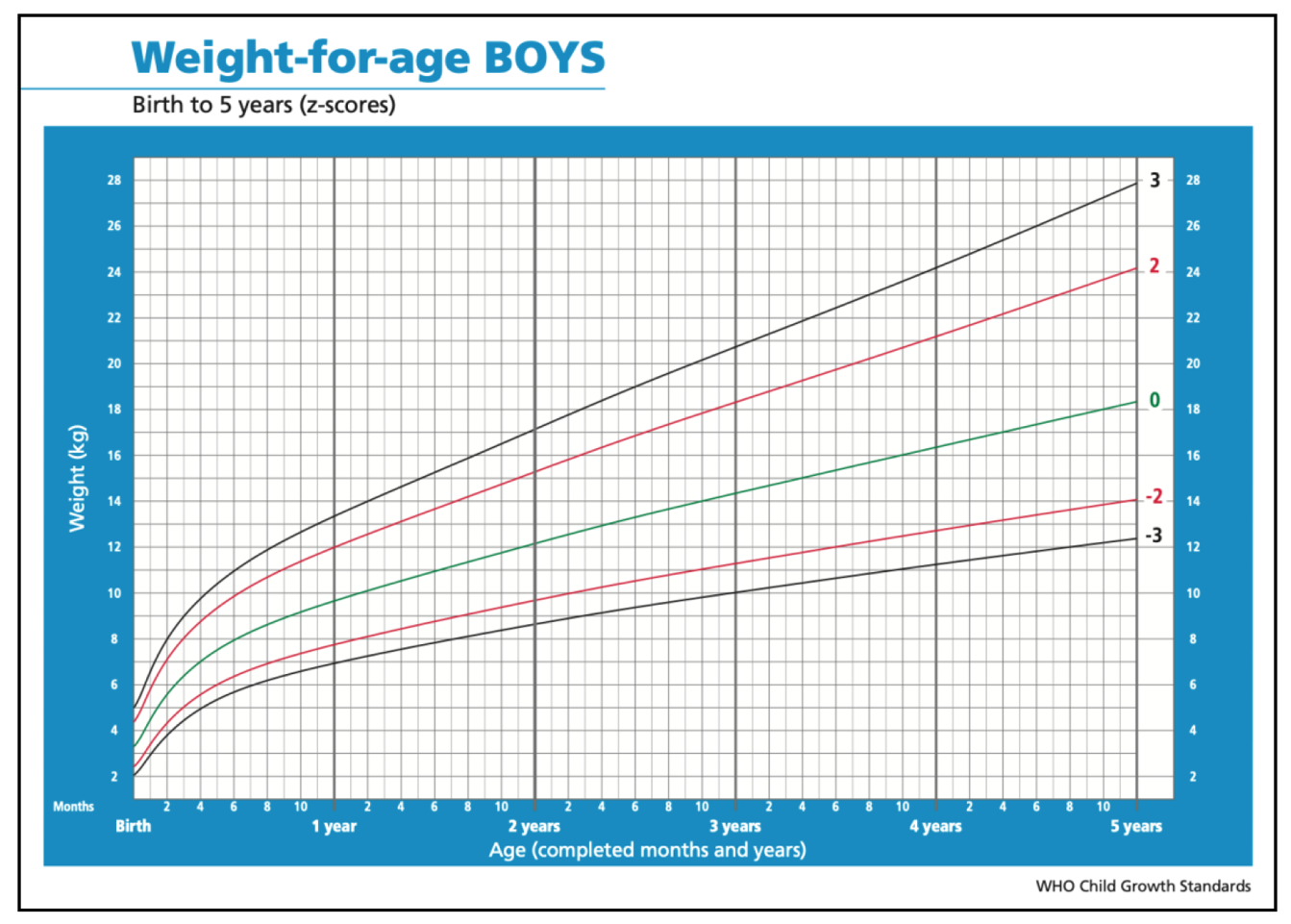


1. WHO weight-for-age look-up table girls' birth to 13 weeks (z score)
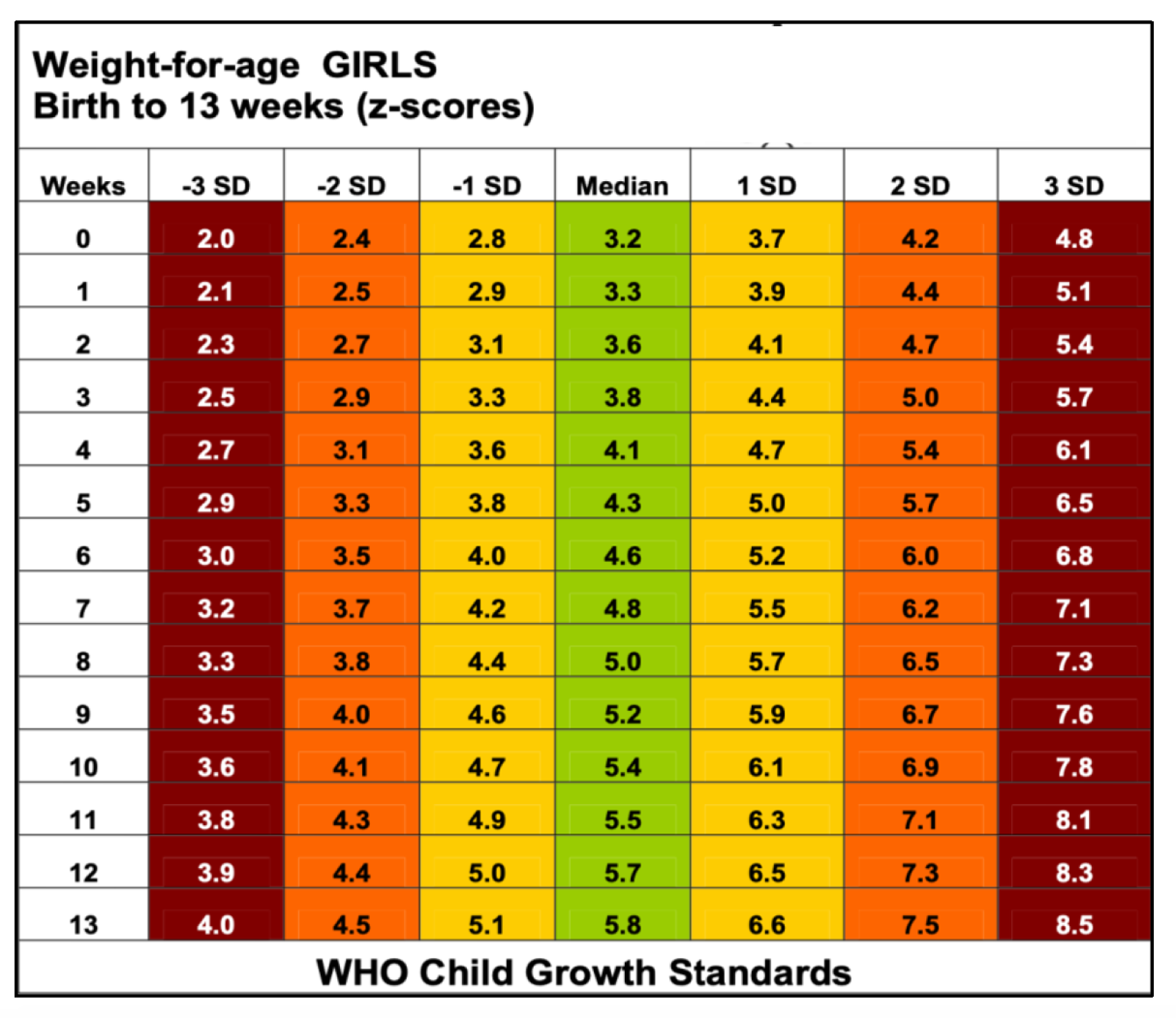

2. WHO weight-for-age look-up table boys' birth to 13 weeks (z scores)
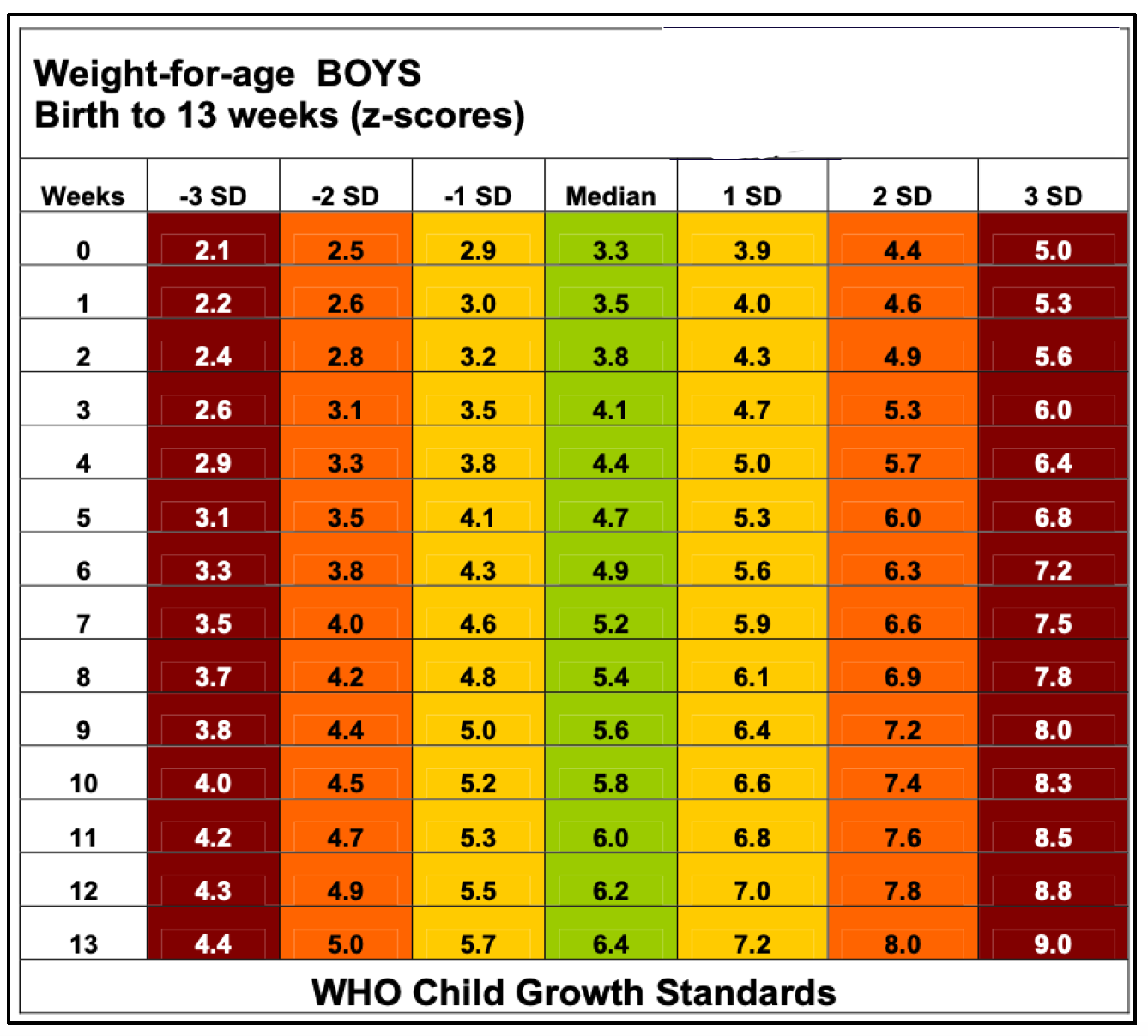

3. WHO weight-for-age look-up table girls' birth to 5 years (z scores)


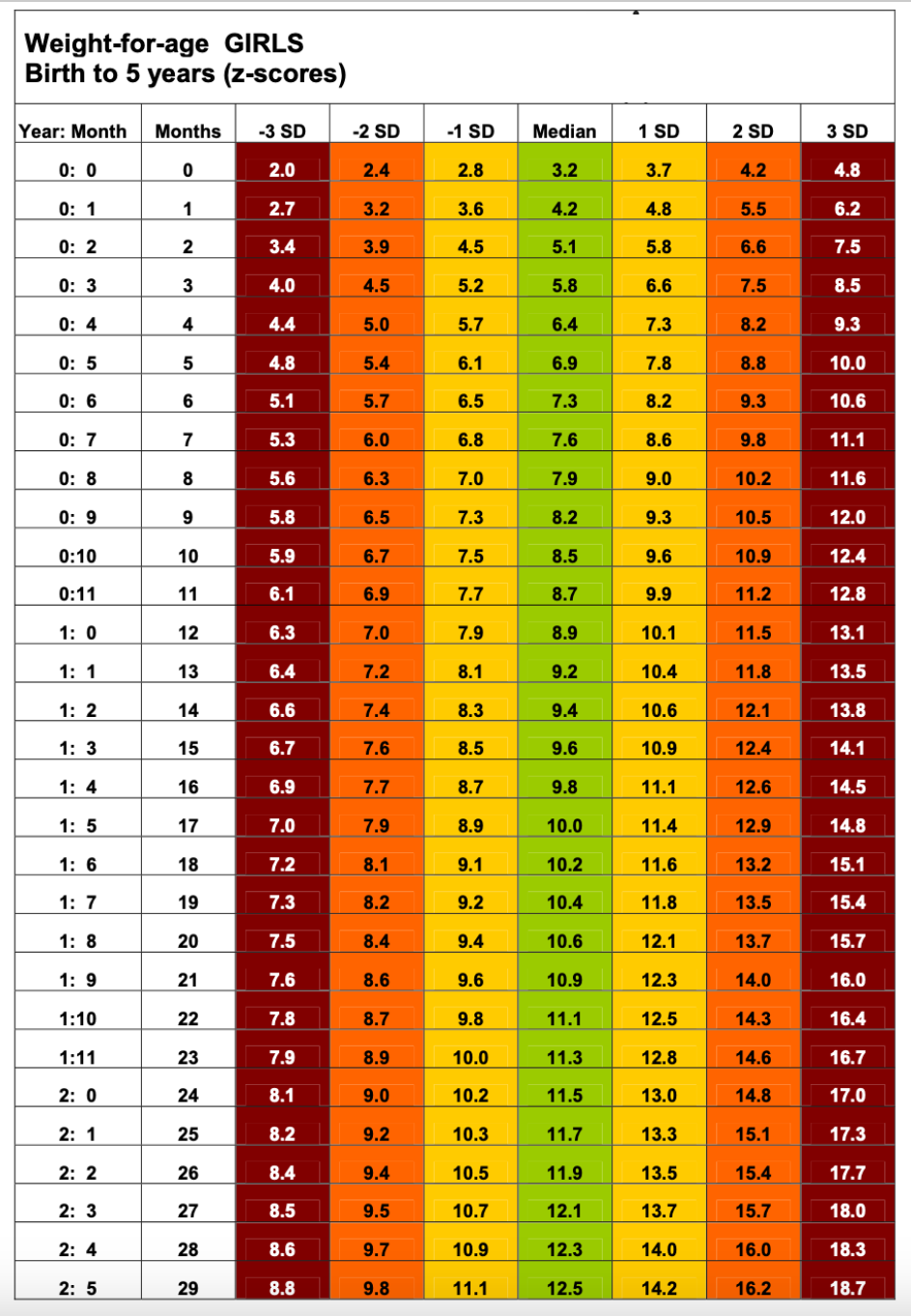


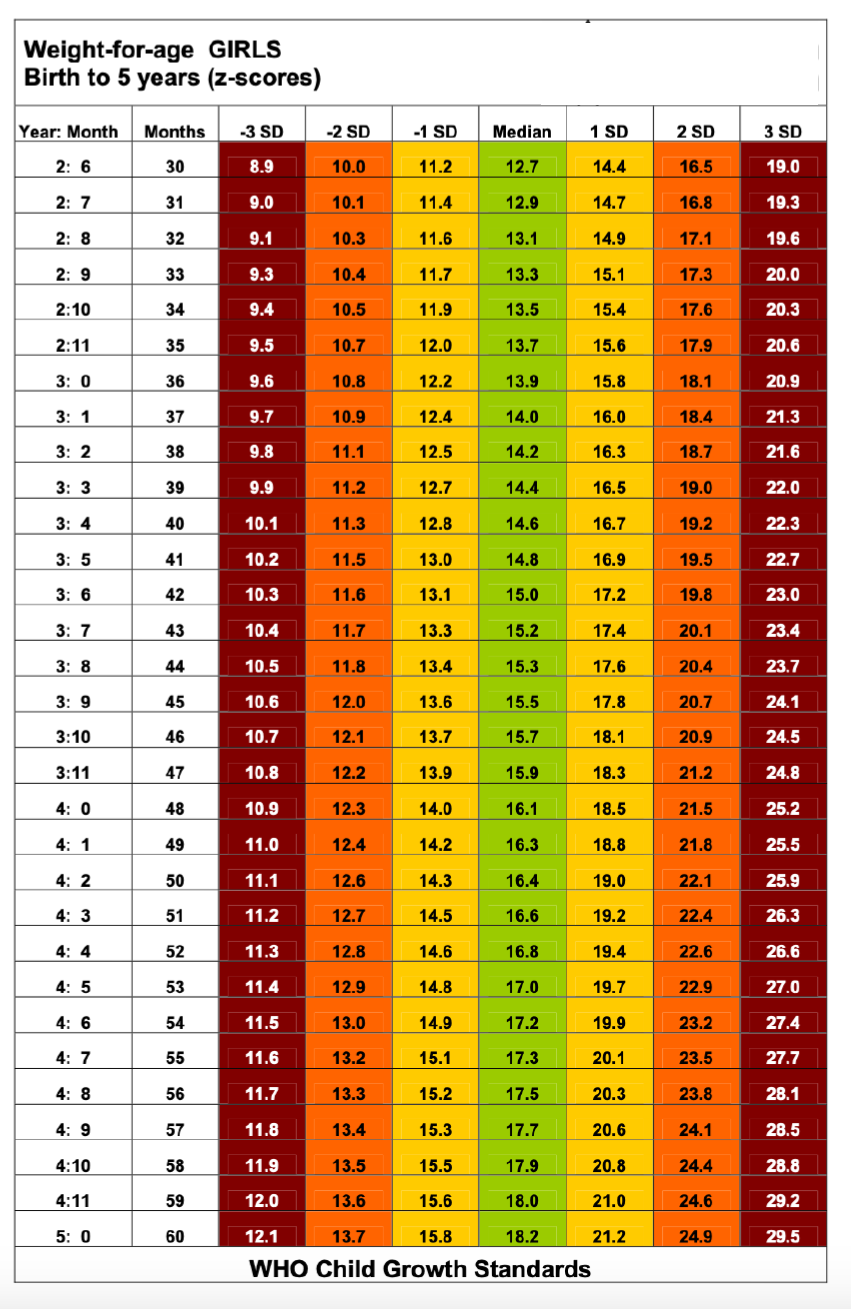


1. WHO weight-for-age look-up table boys' birth to 5 years (z scores)


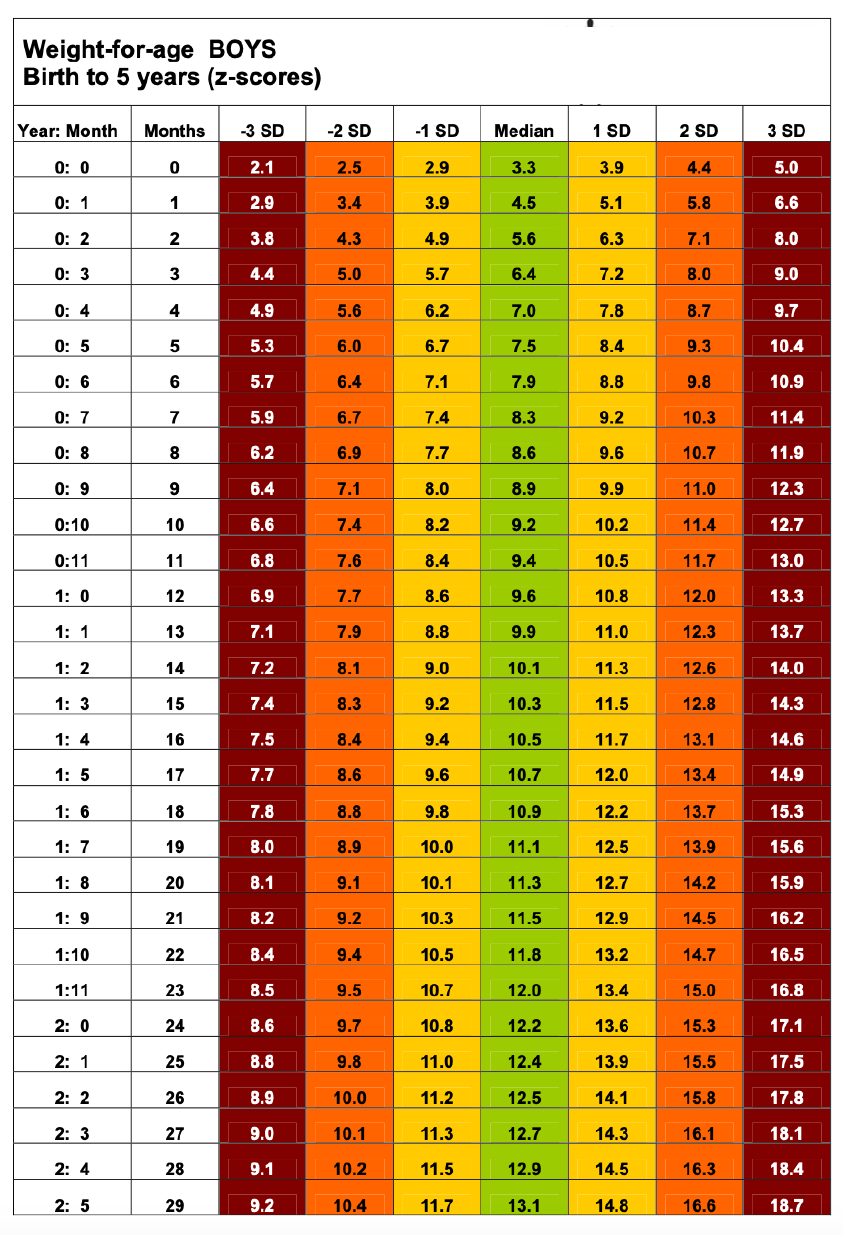


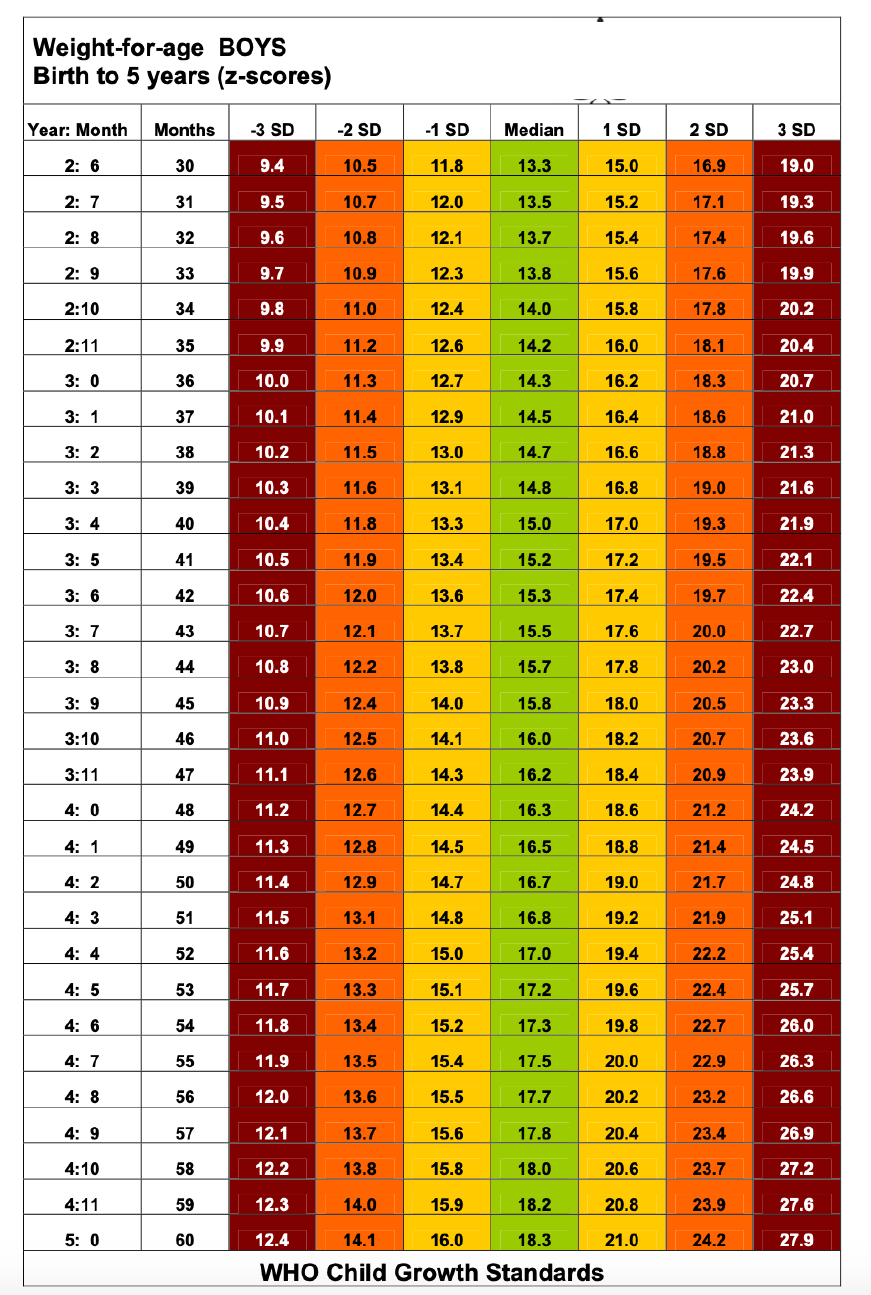

Supplement: S1 Appendix — (DOCX) [file pgph.0002303.s001.docx]
