# Supplementary material for: Identifying underweight in infants and children using growth charts, lookup tables and a novel “MAMI” slide chart: A cross-over diagnostic and acceptability study
Source: PLOS Glob Public Health. 2023 Aug 30;3(8):e0002303. doi: 10.1371/journal.pgph.0002303 (PMC10468082; doi:10.1371/journal.pgph.0002303)
Supplement: S3 Appendix — (DOCX) [file pgph.0002303.s003.docx]

**Design I:** Boys and girls are on either side of the slide chart, 0 to 60 months


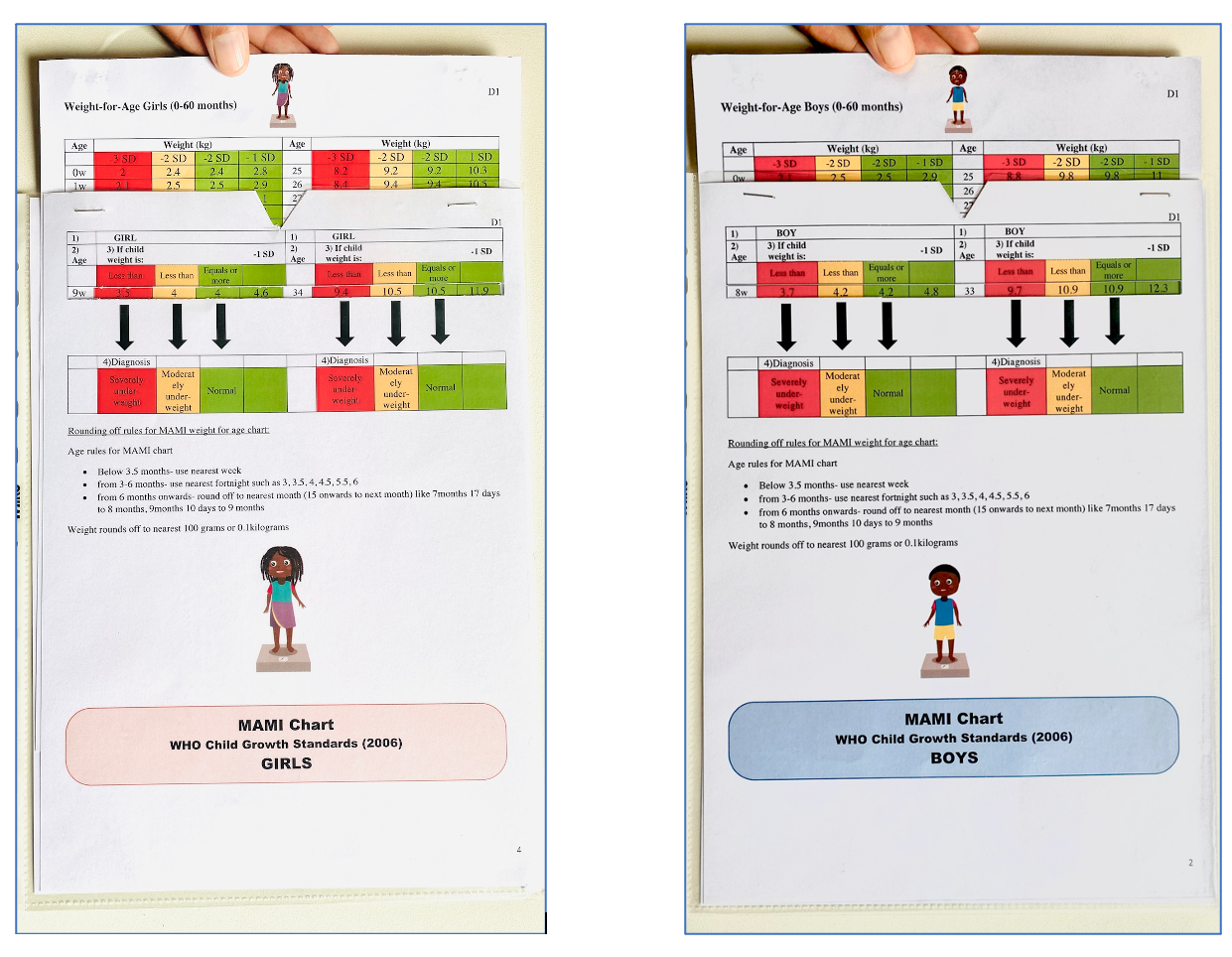


Designed by: Monga, Sikorski, DeSilva, McGrath, Kerac

Designed by: Monga, Sikorski, DeSilva, McGrath, Kerac

**Design II:** Boys and girls are on completely different charts, 0 to 60 months


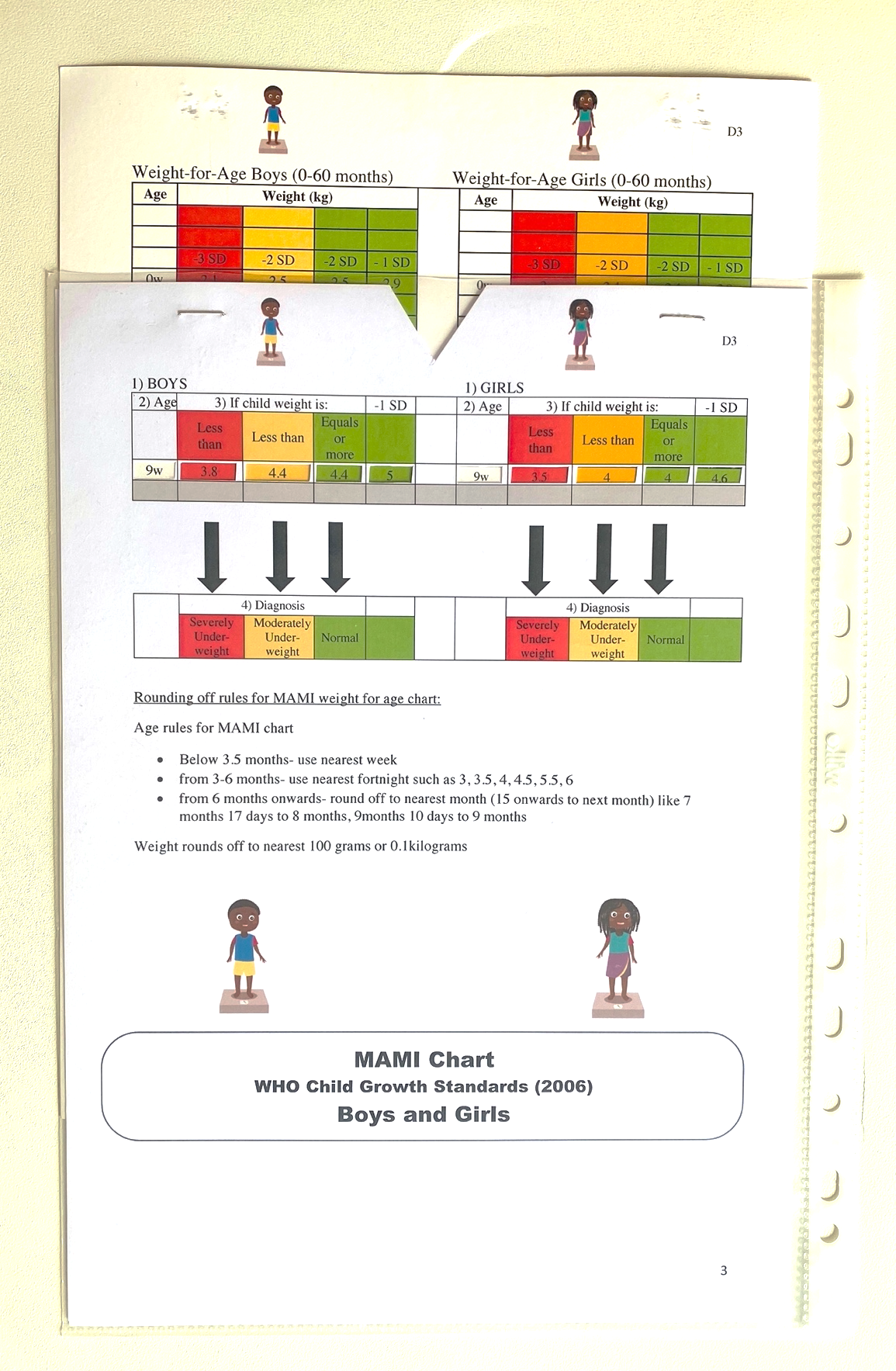


Designed by: Monga, Sikorski, DeSilva, McGrath, Kerac

**
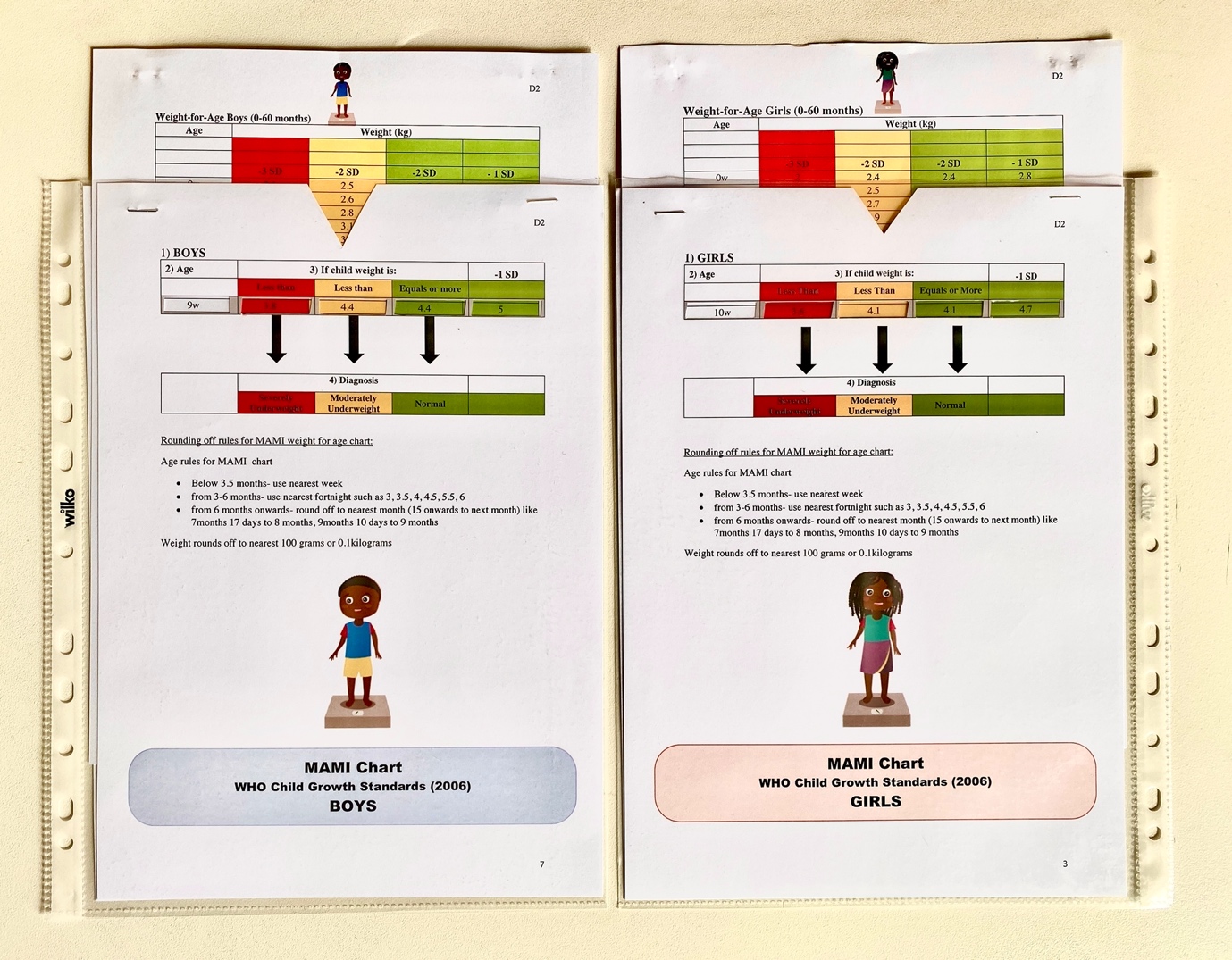
Design III:** Boys and girls are in a side-by-side-parallel arrangement, 0 to 60 months

Designed by: Monga, Sikorski, DeSilva, McGrath, Kerac

Designed by: Monga, Sikorski, DeSilva, McGrath, Kerac
