# Supplementary material for: Identifying underweight in infants and children using growth charts, lookup tables and a novel “MAMI” slide chart: A cross-over diagnostic and acceptability study
Source: PLOS Glob Public Health. 2023 Aug 30;3(8):e0002303. doi: 10.1371/journal.pgph.0002303 (PMC10468082; doi:10.1371/journal.pgph.0002303)
Supplement: S4 Appendix — (DOCX) [file pgph.0002303.s004.docx]

**Step 1:** Confirm whether the child is male or female.

**Step 2:** Round the age to the nearest as per the MAMI chart rounding rules. See rounding rules below:

Rounding off rules for MAMI weight-for-age chart:

Age rules for the MAMI chart

- Below 3.5 months- use the nearest week
- from 3-6 months- use nearest fortnight such as 3, 3.5, 4, 4.5,5.5, 6
- from 6 months onwards- round off to nearest month (15 onwards to next month) like 7months 17 days to 8 months, 9 months 10 days to 9 months

Weight rounds off to the nearest 100 grams or 0.1kilograms

**Step 3:** Slide the chart up and down to find the correct age, and then using the child's weight in kg, check to see if it falls in red, yellow, or green zones. These correspond to thresholds for the severely underweight, moderately underweight or normal category as per weight-for-age criteria.

**Step 4:** use the child's nutritional assessment to refer/treat as per the local guidelines.

Step 3


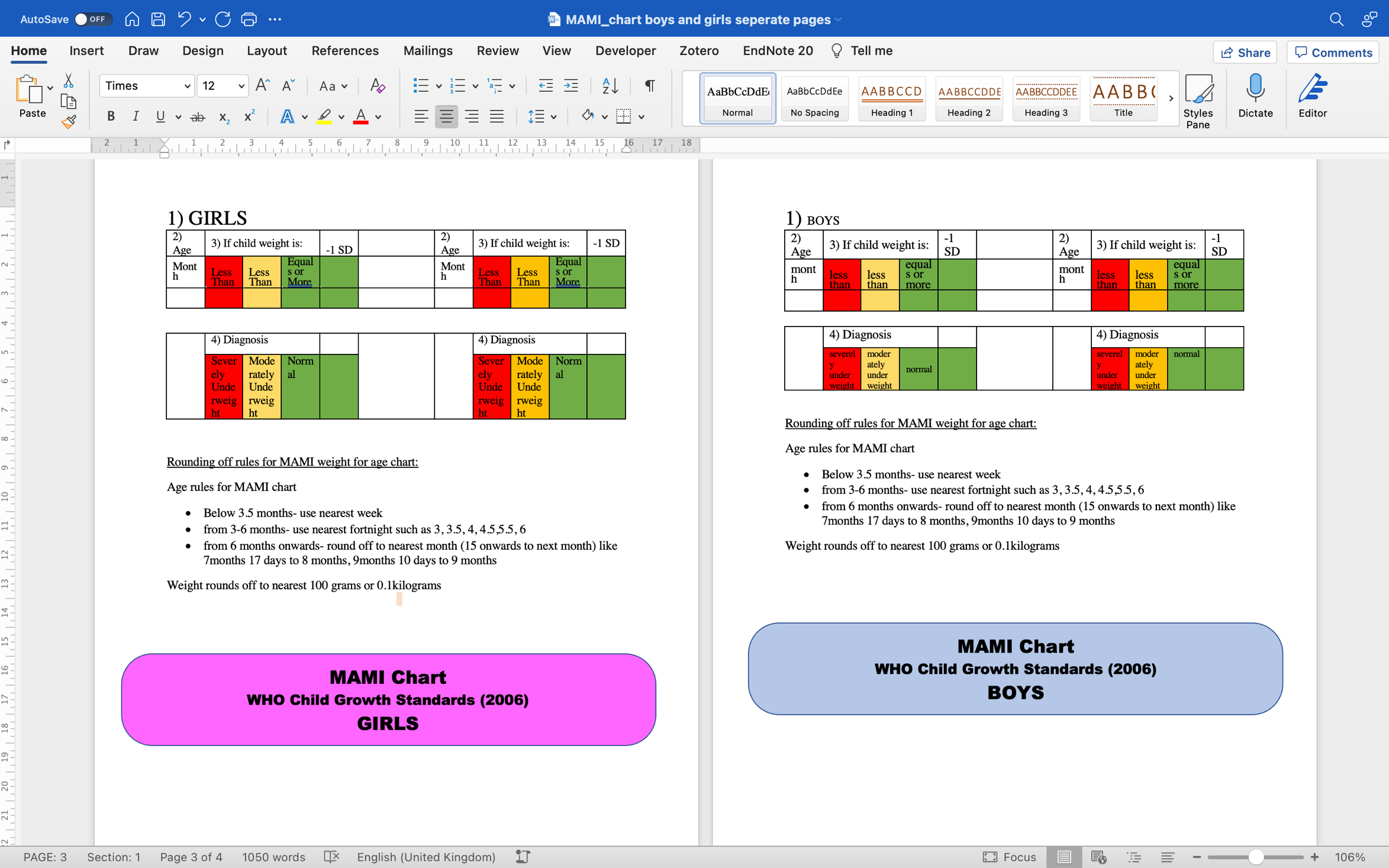


Step 1

Step 2

Step 4

THE MAMI CHART

IMPROVING NUTRITIONAL ASSESSMENT

***What is it:*** The MAMI slide chart is a low-cost tool developed for frontline health care providers to correctly assess the nutritional status of children under five years using weight-for-age criteria.

***Why it has been developed:*** The traditional weight-for-age tables and growth charts for diagnosis of underweight could be difficult to use, interpret and lead to increased chances of errors—resulting in delayed treatment and care. The MAMI chart reduces the possibility of error by an easy-to-use slide design and a step-by-step guide for weight-for-age assessment and interpretation.
