# Supplementary material for: Identifying underweight in infants and children using growth charts, lookup tables and a novel “MAMI” slide chart: A cross-over diagnostic and acceptability study
Source: PLOS Glob Public Health. 2023 Aug 30;3(8):e0002303. doi: 10.1371/journal.pgph.0002303 (PMC10468082; doi:10.1371/journal.pgph.0002303)
Supplement: S5 Appendix — (DOCX) [file pgph.0002303.s005.docx]

Evaluation of a Novel, Low-Cost, Weight-For-Age Slide Chart

Development and Preliminary Evaluation of a Novel, Low-Cost, Weight-For-Age Slide Chart for Improved Nutritional Assessment: A Crossover Pilot Study.

Please answer the following questions by placing a cross or tick in the box provided.

Thank you!

1. Please enter today's date.
2. Please enter the candidate number provided to you
3. Year of experience in Nutrition or Public Health

- 0-5 years
- 5-10 years
- 10-15 years
- Over 15 years

1. Your gender
   - Women
   - Men
   - Non-binary
   - Other
2. Profession
   - Student
   - Researcher
   - Doctor
   - Nurse
   - Mid-wife
   - Other
3. Type of chart you are using to respond
   - MAMI slide chart
   - Look-up table
   - Growth chart
4. *Please write rounded age and diagnosis of the child using information provided below:

Gender: Boy

Age: 5 months 5 days

Weight: 5.2 kg

Write your rounded age in others

- Severely Underweight
- Moderately underweight
- Normal
- Other ________

1. *Please write rounded age and diagnosis of the child using information provided below:

Gender: Boy
Age: 23 days
Weight: 3.1 kg

Write your rounded age in others

- Severely Underweight
- Moderately underweight
- Normal
- Other ________

1. Please write rounded age and diagnosis of the child using information provided below:

Gender: Girl
Age: 4 months 16 days
Weight: 5 kg

Write your rounded age in others

- Severely Underweight
- Moderately underweight
- Normal
- Other ________

1. Please write rounded age and diagnosis of the child using information provided below:

Gender: Boy
Age: 6 months
Weight: 6.6 kg

Write your rounded age in others

- Severely Underweight
- Moderately underweight
- Normal
- Other ________

1. *Please write rounded age and diagnosis of the child using information provided below:

Gender: Girl
Age: 3 years 2 months
Weight: 9.8 kg

Write your rounded age in others

- Severely Underweight
- Moderately underweight
- Normal
- Other ________

1. Please write rounded age and diagnosis of the child using information provided below:

Gender: Boy
Age: 2 years
Weight: 8.5 kg

Write your rounded age in others

- Severely Underweight
- Moderately underweight
- Normal
- Other ________

1. Please write rounded age and diagnosis of the child using information provided below:

Gender: Girl
Age: 4 years 9 months
Weight: 11.9 kg

Write your rounded age in others

- Severely Underweight
- Moderately underweight
- Normal
- Other ________

1. Please write rounded age and diagnosis of the child using information provided below:

Gender: Boy
Age: 42 days
Weight: 3.6 kg

Write your rounded age in others

- Severely Underweight
- Moderately underweight
- Normal
- Other ________

1. Please write rounded age and diagnosis of the child using information provided below:

Gender: Boy
Age: 1 years 9 months
Weight: 10.5 kg

Write your rounded age in others

- Severely Underweight
- Moderately underweight
- Normal
- Other ________

1. *Please write rounded age and diagnosis of the child using information provided below:

Gender: Boy
Age: 4 years 3 months
Weight: 13.1 kg

Write your rounded age in others

- Severely Underweight
- Moderately underweight
- Normal
- Other ________

1. Please write rounded age and diagnosis of the child using information provided below:

Gender: Boy
Age: 1 years 1 month
Weight: 7.5 kg

Write your rounded age in others

- Severely Underweight
- Moderately underweight
- Normal
- Other ________

1. Please write rounded age and diagnosis of the child using information provided below:

Gender: Girl
Age: 15 days
Weight: 2.5 kg

Write your rounded age in others

- Severely Underweight
- Moderately underweight
- Normal
- Other ________

1. *Please write rounded age and diagnosis of the child using information provided below:

Gender: Boy
Age: 35 days
Weight: 3.5 kg

Write your rounded age in others

- Severely Underweight
- Moderately underweight
- Normal
- Other ________

1. Please write rounded age and diagnosis of the child using information provided below:

Gender: Girl
Age: 50 days
Weight: 3.6 kg

Write your rounded age in others

- Severely Underweight
- Moderately underweight
- Normal
- Other ________

1. *Please write rounded age and diagnosis of the child using information provided below:

Gender: Girl
Age: 2 years 2 months
Weight: 8.4 kg

Write your rounded age in others

- Severely Underweight
- Moderately underweight
- Normal
- Other ________

1. Please write rounded age and diagnosis of the child using information provided below:

Gender: Boy
Age: 50 days
Weight: 3.5 kg

Write your rounded age in others

- Severely Underweight
- Moderately underweight
- Normal
- Other ________

1. Please write rounded age and diagnosis of the child using information provided below:

Gender: Girl
Age: 4 years 4 months
Weight: 12.5 kg

Write your rounded age in others

- Severely Underweight
- Moderately underweight
- Normal
- Other ________

1. Please write rounded age and diagnosis of the child using information provided below:

Gender: Boy
Age: 2 years 9 months
Weight: 8.1 kg

Write your rounded age in others

- Severely Underweight
- Moderately underweight
- Normal
- Other ________

1. *Please write rounded age and diagnosis of the child using information provided below:

Gender: Girl
Age: 2 years 9 months
Weight: 9.3 kg

Write your rounded age in others

- Severely Underweight
- Moderately underweight
- Normal
- Other ________

1. Please write rounded age and diagnosis of the child using information provided below:

Gender: Boy
Age: 7 months 15 days
Weight: 5.9 kg

Write your rounded age in others

- Severely Underweight
- Moderately underweight
- Normal
- Other ________

1. Please write rounded age and diagnosis of the child using information provided below:

Gender: Boy
Age: 25 months
Weight: 8.7 kg

Write your rounded age in others

- Severely Underweight
- Moderately underweight
- Normal
- Other ________

1. Please write rounded age and diagnosis of the child using information provided below:

Gender: Girl
Age: 59 months 14 days
Weight: 13.7 kg

Write your rounded age in others

- Severely Underweight
- Moderately underweight
- Normal
- Other ________

1. Please write rounded age and diagnosis of the child using information provided below:

Gender: Girl
Age: 10 weeks 4 days
Weight: 3.5 kg

Write your rounded age in others

- Severely Underweight
- Moderately underweight
- Normal
- Other ________

1. Please write rounded age and diagnosis of the child using information provided below:

Gender: Boy
Age: 34 months 23 days
Weight: 11.1 kg

Write your rounded age in others

- Severely Underweight
- Moderately underweight
- Normal
- Other ________

1. *Please write rounded age and diagnosis of the child using information provided below:
   Gender: Girl
   Age: 1 years 2 months
   Weight: 6.6 kg

Write your rounded age in others

- Severely Underweight
- Moderately underweight
- Normal
- Other ________

Note: '*' marked hypothetical scenarios have borderline weight, i.e., weight-for-age z scores is exactly at -2 or -3 z score.
