# Supplementary material for: Identifying underweight in infants and children using growth charts, lookup tables and a novel “MAMI” slide chart: A cross-over diagnostic and acceptability study
Source: PLOS Glob Public Health. 2023 Aug 30;3(8):e0002303. doi: 10.1371/journal.pgph.0002303 (PMC10468082; doi:10.1371/journal.pgph.0002303)
Supplement: S6 Appendix — (DOC) [file pgph.0002303.s006.doc]

Questionnaire aims to assess the weight-for-age MAMI chart and to determine how easy people find using it? Please answer the following questions by placing a cross or tick in box provided. Thank you!

| Candidate no. _______________ Date: ­__/____/_2022 |
| --- |

1. How easy or difficult is it to use the MAMI chart compared to the Look-up table?

The MAMI chart is...

Much more difficult Slightly more difficult About the same Slightly easier Much easier

🞏 🞏 🞏 🞏 🞏

2. How easy or difficult is it to use the MAMI chart compared to the Traditional growth chart?

The MAMI chart is...

Much more difficult Slightly more difficult About the same Slightly easier Much easier

🞏 🞏 🞏 🞏 🞏

3. How much faster or slower is it when you use the MAMI chart?

The MAMI chart is...

Much faster Slightly faster About the same Slightly slower Much slower

🞏 🞏 🞏 🞏 🞏

4. When using the look up tables, how often do you need to make corrections?

Never Rarely Sometimes Often All the time

🞏 🞏 🞏 🞏 🞏

5. When using the traditional growth chart, how often do you need to make corrections?

Never Rarely Sometimes Often All the time

🞏 🞏 🞏 🞏 🞏

6. When using the MAMI chart, how often do you need to make corrections?

Never Rarely Sometimes Often All the time

🞏 🞏 🞏 🞏 🞏

7. How stressful do you find using the Look-up table?

Not stressful Slightly stressful Moderately stressful Highly stressful

🞏 🞏 🞏 🞏

8. How stressful do you find using the traditional growth chart?

Not stressful Slightly stressful Moderately stressful Highly stressful

🞏 🞏 🞏 🞏

9. How stressful do you find using the MAMI chart?

Not stressful Slightly stressful Moderately stressful Highly stressful

🞏 🞏 🞏 🞏

10. Which method of diagnosis would you prefer to use if you were working in a nutrition clinic?

MAMI chart Look-up table Traditional Growth Chart

🞏 🞏 🞏

11. Have you ever used a tool like the MAMI chart before?

Never Rarely Occasionally Often

🞏 🞏 🞏 🞏

12. Have you ever used a tool like the look up tables before?

Never Rarely Occasionally Often

🞏 🞏 🞏 🞏

13. Have you ever used a tool like the traditional growth chart before?

Never Rarely Occasionally Often

🞏 🞏 🞏 🞏

14. Out of three MAMI chart design shown, which one do you prefer the most?

Design 1 Design 2 Design 3

🞏 🞏 🞏

15. How could we *improve* the MAMI chart to make it easier to use? (e.g., design, format, instructions...)

......................................................................................................................................................................................................................................................................................................................................................................................................................................................................................................
